# Antimicrobial Effects of Sulfonyl Derivative of 2(5*H*)-Furanone against Planktonic and Biofilm Associated Methicillin-Resistant and -Susceptible *Staphylococcus aureus*

**DOI:** 10.3389/fmicb.2017.02246

**Published:** 2017-11-20

**Authors:** Irshad S. Sharafutdinov, Elena Y. Trizna, Diana R. Baidamshina, Maria N. Ryzhikova, Regina R. Sibgatullina, Alsu M. Khabibrakhmanova, Liliya Z. Latypova, Almira R. Kurbangalieva, Elvira V. Rozhina, Mareike Klinger-Strobel, Rawil F. Fakhrullin, Mathias W. Pletz, Mikhail I. Bogachev, Airat R. Kayumov, Oliwia Makarewicz

**Affiliations:** ^1^Institute of Fundamental Medicine and Biology, Kazan Federal University, Kazan, Russia; ^2^Biofunctional Chemistry Laboratory, Alexander Butlerov Institute of Chemistry, Kazan Federal University, Kazan, Russia; ^3^Center for Infectious Diseases and Infection Control, Jena University Hospital, Jena, Germany; ^4^Biomedical Engineering Research Centre, Saint Petersburg Electrotechnical University, Saint Petersburg, Russia

**Keywords:** 2(5*H*)-furanones, sulfones, *Staphylococcus aureus*, MRSA, biofilm, antimicrobials synergism

## Abstract

The gram-positive opportunistic bacterium *Staphylococcus aureus* is one of the most common causatives of a variety of diseases including skin and skin structure infection or nosocomial catheter-associated infections. The biofilm formation that is an important virulence factor of this microorganism renders the antibiotic therapy ineffective, because biofilm-embedded bacteria exhibit strongly increased tolerance to antimicrobials. Here, we describe a novel 3-chloro-5(*S*)-[(1*R*,2*S*,5*R*)-2-isopropyl-5-methylcyclohexyloxy]-4-[4-methylphenylsulfonyl]-2(5*H*)-furanone (**F105**), possessing a sulfonyl group and *l*-menthol moiety. Minimal inhibitory and bactericidal concentration values (MIC and MBC) of **F105** were 10 and 40 mg/L, respectively, suggesting **F105** biocidal properties. **F105** exhibits pronounced activity against biofilm-embedded *S. aureus* and increases the efficacy of aminoglycosides (amikacin, gentamicin, and kanamycin) and benzalkonium chloride with fractional inhibitory concentration index values of 0.33–0.44 and 0.29, respectively, suggesting an alternative external treatment option, e.g., for wound infections. Moreover, low concentrations (0.5–1.3 mg/L) of **F105** reduced the MICs of these antimicrobials twofold. By using confocal laser scanning microscopy and CFU counting, we show explicitly that **F105** also restores the antimicrobial activity of gentamicin and ampicillin against *S. aureus* biofilms by several orders of magnitude. Biofilm structures were not destroyed but sterilized, with embedded cells being almost completely killed at twofold MBC. While **F105** is quite toxic (CC_50_/MBC ratio 0.2), our data suggest that the **F105** chemotype might be a promising starting point for the development of complex topical agents for combined anti-staphylococcal biofilm-therapies restoring the efficacy of some antibiotics against difficult to treat *S. aureus* biofilm.

## Introduction

Formation of biofilms represents an important virulence factor of the Gram-positive opportunist *Staphylococcus aureus* (McCarthy et al., 2015; Naicker et al., 2016), one of the most common causatives of a variety of biofilm-associated diseases such as osteomyelitis, endocarditis, skin and skin structure infection (SSSI) as well as foreign body associated infections, commonly leading to the development of sepsis (Conlon, 2014). Therefore, infections caused by *S. aureus* are associated with increased morbidity and mortality (Bassetti et al., 2017).

Biofilms are complex three-dimensional microbial communities attached to a multitude of surfaces representing the preferred life-style of the bacteria in natural and artificial habitats (Donlan and Costerton, 2002; Bremer et al., 2011). In biofilms, bacterial cells are embedded in an extracellular matrix of organic polymers such as polysaccharides, peptides, and extracellular DNA that are synthesized and released by the microbes themselves (Lewis, 2001; Atshan et al., 2015; Fagerlund et al., 2016). The matrix drastically reduces the susceptibility to different outer stress factors (Donlan, 2002) indicated by up to 1000-fold higher tolerance to antimicrobials of the biofilm-embedded cells compared to their planktonic counterparts (Cosgrove et al., 2002; Sanchez-Vizuete et al., 2015). Treatment of chronic infections is often complicated due to the presence of bacterial biofilm on the surface of the wound that detain the healing process (Doll et al., 2016; Sharafutdinov et al., 2016; Roy et al., 2017). In addition, biofilms demonstrate increased robustness against the host immune system leading to recurrent and/or persistent chronic infections. In addition, the genotypic resistance of bacteria can increase the overall antimicrobial resistance of the biofilm-embedded bacteria providing the so called herd-protection for the susceptible co-occupants. The conjugative acquisition of methicillin resistance is also a strong limiting factor for the antimicrobial therapy efficacy of *S. aureus* caused infections. Infections by methicillin-resistant *S. aureus* strains (MRSA) have even poorer outcomes (Cosgrove and Fowler, 2008) requiring alternative therapeutic options.

In general, only very few antibiotics are capable of directly attacking bacterial biofilms. For example, rifampicin in combination with other antibiotics such as β-lactam is often used against persisting staphylococcal biofilms (Forrest and Tamura, 2010), because bacteria rapidly develop rifampicin resistance under treatment. Experimental data also suggest that ribosome-active antibiotics (such as linezolid or clindamycin) might be effective against staphylococcal biofilms because of their comparable suppressive effect on the expression of various virulence factors (Hodille et al., 2017). However, clinical evidence is still missing; thus, nowadays neither linezolid nor clindamycin are recommended for treatment of biofilm-associated staphylococcal infections.

Investigations on alternative treatment options against biofilm-associated infections is largely based upon the use of specialized agents (such as quaternary ammonium compounds, curcumin or chlorquinaldol) that in combinations with antibiotics provide high local drug concentrations avoiding systemic adverse effects (Kali et al., 2016; Percival et al., 2016; Bortolin et al., 2017). Among various compounds exhibiting antimicrobial and anti-biofilm activities, the derivates of 2(5*H*)-furanone have been intensively studied in last two decades. In the nature, furanones are known to exhibit many different functions, such as intra- and inter-species signaling and communication, attractant molecules and pheromones, antimicrobials, and anti-carcinogens (Fenske and Merzweiler, 1989; Donlan and Costerton, 2002; Bremer et al., 2011). Several studies have reported the ability of synthetic furanones to inhibit biofilm formation of various bacteria (Ren et al., 2001; Hentzer et al., 2002; Lonn-Stensrud et al., 2009; Fedorova et al., 2013). While many 2(5*H*)-furanone derivatives interfere with AI-II quorum-sensing systems of Gram-negative bacteria thereby blocking the biofilm growth (Ren et al., 2001), a number of furanones were shown to repress the biofilm formation by *Bacillus subtilis* and *Staphylococci* (Heck and Stuetz, 1988; Ren et al., 2004; Lonn-Stensrud et al., 2009; Kayumov et al., 2015a). In particular, Lonn-Stensrud et al. (2009) reported that (*Z*)-5-(bromomethylene)furan-2(5*H*)-one completely repressed the biofilm formation by *S. epidermidis* without any irritative or genotoxic effects. In contrast, brominated furanone increased the production of the extracellular matrix by *S. aureus* (Yujie et al., 2013) indicating no universality in the effects of these compounds on bacterial cells. Besides the biofilm repression effects, in several other studies furanone derivatives were reported to exhibit bactericidal activity against gram positive bacteria (Ren et al., 2004; Kitty et al., 2015).

It has been previously shown that the introduction of *l*-menthol moiety into carbamate derivatives significantly increases their anti-biofilm properties toward multidrug-resistant *S. aureus* (Rogers et al., 2010). Several other studies reported that sulfonyl-containing compounds efficiently repressed the growth and biofilm formation by *Staphylococci* (Meadows and Gervay-Hague, 2006; Kudryavtsev et al., 2009; Low et al., 2011). Therefore, we aimed to investigate the antimicrobial activity of a novel 2(5*H*)-furanone derivative (**F105**) possessing two pharmacophores, a sulfonyl group and an *l*-menthol moiety. We found synergy of **F105** with aminoglycosides against planktonic *S. aureus* and demonstrated their attractive activity toward the biofilm-embedded bacteria.

## Materials and Methods

### Chemistry

3-Chloro-5(*S*)-[(1*R*,2*S*,5*R*)-2-isopropyl-5-methylcyclohexyloxy]-4-[4-methylphenylsulfonyl]-2(5*H*)-furanone (**F105**) was synthesized in three steps. For the detailed description of compounds preparation and characterization, we refer to the Supplementary Data, available online. The stock solutions of **F105** were prepared by diluting powders in pure DMSO (Sigma–Aldrich, Saint-Quentin Fallavier, France) at the concentration of 20 g/L. To solubilize the furanone at high concentrations in medium, pluoronic acid F-127 (Sigma–Aldrich) (10% stock solution in DMSO) was added to the final concentration of 0.1%. Working solutions were prepared in bacterial growth medium such that the final concentrations of DMSO 5% have been obtained, which was next verified to be non-toxic for the bacterial strains tested. All conventional antibiotics were purchased from Sigma.

### Strains and Culture Conditions

A methicillin sensitive *Staphylococcus aureus* ATCC^®^29213 (MSSA) and a methicillin resistant *S. aureus* strain ATCC^®^43300 (MRSA) (both laboratory strains) as well as 10 clinical MRSA isolates provided by the Republican Clinical Hospital, Laboratory of Clinical Bacteriology in Kazan were used in this study (see **Table 1**). The bacterial strains were stored in 10% (V/V) glycerol stocks at -80°C and freshly streaked on blood agar plates (BD Diagnostics) following by their overnight growth at 35°C before use. Fresh colony material was used to adjust an optical density to 0.5 McFarland (equivalent to 10^8^ cells/mL) in 0.9% NaCl solution that was used as a working suspension.

**Table 1:** The antimicrobial effects of **F105** expressed as MIC, MBC, BPC and MBEC against *S. aureus* isolates in mg/L.

*S. aureus* strain	MIC	MBC	BPC	MBEC
ATCC 29213 (MSSA)	10	40	40 (40^∗^)	80 (160^∗^)
ATCC 43300 (MRSA)	20	80	80 (40^∗^)	80^∗^
*S. aureus* 1053	10	20	20	n.d.^∗∗^
*S. aureus* 1130	10	40	20	n.d.
*S. aureus* 1131	10	20	20	n.d.
*S. aureus* 1134	10	20	20	n.d.
*S. aureus* 1145	10	40	20	n.d.
*S. aureus* 1163	10	40	40	n.d.
*S. aureus* 1167	10	20	20	n.d.
*S. aureus* 1168	10	20	20	n.d.
*S. aureus* 1173	10	40	20	n.d.
*S. aureus* 2020	10	40	20	n.d.


*MIC and MBC were assessed by the micro dilution method, BPC and MBEC by the drop plate method or by CLSM^∗^.*

*^∗^The values in parentheses represent the concentration at which biofilm formation was visibly inhibited in CLSM-analysis.*

*^∗∗^n.d., not determined.*

### Determination of the Minimal Inhibitory (MIC) and the Minimal Bactericidal Concentrations (MBC) of F105

The MIC of furanone **F105** was determined by the broth microdilution method in 96-well microtiter plates (Eppendorf) according to the EUCAST rules for antimicrobial susceptibility testing (Leclercq et al., 2013) with minor modifications to account for the decreased solubility of the compound. Briefly, the 10^8^ cells/mL bacterial suspension was subsequently diluted 1:300 with Mueller-Hinton broth (MH) (Carl Roth GmbH, Germany), cation-adjusted with 20 mg/L Ca^2+^ and 10 mg/L Mg^2+^ and supplemented with various concentrations of **F105** in microwell plates to obtain a 3 × 10^5^ cells/mL suspension. To solubilize the furanone at high concentrations in medium, pluoronic acid F-127 (Sigma–Aldrich) (10% stock solution in DMSO) was added to the final concentration of 0.1%. The final concentration of DMSO was adjusted to 5% in all bacterial cultures. The concentrations of **F105** ranged from 1.25 to 160 mg/L. Besides the usual double dilutions, additional concentrations were included in between. The cultures were incubated at 35°C for 24 h. The MIC was determined as the lowest concentration of furanone for which no visible bacterial growth could be observed after 24 h of incubation.

To determine the MBC, the CFU/mL were further evaluated in culture liquid from wells without visible growth. The **F105** concentration reducing the number of viable cells by at least three orders of magnitude was considered as MBC according to the recommendation of the European Committee for Antimicrobial Susceptibility Testing (EUCAST) of the European Society of Clinical Microbiology and Infectious Diseases (ESCMID) (2000).

### Synergy Testing by Checkerboard Assay

The checkerboard assay was performed similarly to the MIC testing in 96-well microtiter plates (Eppendorf). Each plate contained serial dilutions of **F105** in 0.1% pluoronic acid F-127 and different antibiotics in a checkerboard fashion as described previously (Eliopoulos and Moellering, 1996). Briefly, the final concentrations of both compounds ranged from 1/16× to 4× MIC for **F105** and from 1/256× to 4× MIC for the antibiotics. In total, 11 dilution steps of antibiotics and 7 dilution steps of **F105** were analyzed. The microwell plates were incubated at 35°C for 24 h. Each test was performed in triplicate and included a growth control with neither antibiotic nor **F105** addition. The fractional inhibitory concentration index (FICI) for each double combination was calculated as

$${FICI}_{Antibiotic/F105}=\frac{{MIC}_{Antibiotic\,\;\left(combination\right)}}{{MIC}_{Antibiotic\,\;\left(alone\right)}}+\frac{{MIC}_{F105\,\;\left(combination\right)}}{{MIC}_{F105\,\;\left(alone\right)}}.$$

The FICIs were counted from the concentrations in the first non-turbid well found in each row and column along the turbidity/non-turbidity interface and the lowest FICI value was used to characterize the synergy. For the FICI interpretation we refer to den Hollander et al. (1998) and Odds (2003): FICI < 0.5 corresponds to synergy, 0.5 < FICI < 4 corresponds to either additive effects or indifference, while FICI > 4 corresponds to antagonism.

### Analysis of the Biofilm Prevention Concentration (BPC) of F105

To determine the BPC of **F105**, two methods have been applied, including the modified crystal-violet staining (Merritt et al., 2005) and the drop plate approach (Herigstad et al., 2001). Briefly, the bacterial culture was adjusted to 5 × 10^5^ cells/mL in the MH broth and seeded into 24-well polystyrene culture plates (Eppendorf). **F105** was added in serial dilutions to the final concentrations between 1.25 and 160 mg/L following by the cells growth under static conditions for 24 h at 35°C. For the crystal violet staining, the liquid culture was removed after 24 h of incubation and the plates were washed twice with PBS (pH 7.4) and dried. Then 1 ml of the 0.5% crystal violet solution (Sigma) in 96% ethanol was added per well, followed by 20 min incubation. Next the crystal violet solution was removed and the plate was washed 3 times with PBS. After 30 min air drying, 1 ml of 96% ethanol was added to resolubilize the bound crystal violet, and the absorbance was measured at 570 nm with the microplate reader Infinite 200 Pro (Tecan). Cell-free wells incubated with pure medium subjected to all staining manipulations were used as control.

Alternatively, the viability of cells was evaluated by the drop plate approach with minor modifications (Herigstad et al., 2001). Serial 10-fold dilutions from each well were prepared and 5 ml of suspension was dropped onto LB agar plates in five repeats. CFU/mL were counted and averaged from those drops containing 5–30 colonies. To evaluate the viability of biofilm-embedded cells, wells were washed several times with phosphate-buffered saline (PBS) to remove both non-adherent and detached cells. The washed biofilms were suspended in PBS by scratching the well bottoms with following treatment in a sonicator bath for 2 min at 20 kHz to favor the disintegration of bacterial clumps, and viable cells were counted by the drop plate method as described above.

#### Time-Kill Assay

Time-kill curves were obtained by growing MSSA in MH medium in the presence of **F105** at concentrations of 0.5× MBC, 1× MBC, and 2× MBC. The starting bacterial suspension was adjusted to the 5 × 10^7^ cells/mL concentration. Cells were then cultivated in 6-well microtiter plates without shaking at 35°C for 12 h and samples were taken every 4 h, serially diluted 1:10 and plated onto agar plates. The CFU/mL was counted after incubation at 35°C for at least 16 h.

#### Determination of Anti-biofilm Activity of F105 by CLSM

To additionally compare the BPC of MSSA and MRSA standard strains by a microscopic method, the biofilms were grown in the presence of **F105** as descried above. To determine the minimal biofilm eradicating concentration (MBEC), the biofilms were grown without **F105** under static conditions for 24 h at 35°C. Subsequently, the supernatants were carefully removed and the biofilms were treated with 500 μL of **F105** solutions with various concentrations between 5 and 160 mg/L serially diluted in MH broth, and cultivation was continued for 24 h at 35°C. The effect of ampicillin, gentamicin, and benzalkonium chloride on the biofilm-embedded cells was analyzed similarly.

For both BPC and MBEC determination, the liquid cultures were carefully removed and the biofilms were washed once with 300 μl of 0.9% NaCl solution. The biofilms were stained using LIVE/DEAD BacLight Bacterial Viability Kit for microscopy (Life Technologies GmbH) according to manufacturer’s protocol. Stained biofilms were analyzed under vital conditions using an inverted confocal laser scanning microscope (CLSM) Carl Zeiss LSM 780 (Carl Zeiss AG) at green (522 nm) and red (635 nm) filters, respectively, using laser excitation at 490 nm as described previously (Klinger-Strobel et al., 2016; Baidamshina et al., 2017). An area of approximately 100 μm (X) × 100 μm (Y) was screened in 1 μm Z-intervals (Z-stack). The biofilm microscopy data were processed using ZEN 9.0 software (Carl Zeiss AG).

The BPC was considered as the furanone concentration at which the complete absence of the biofilm assessed with crystal violet staining was observed (Peeters et al., 2008). **F105** concentrations reducing the viable cells by at least 3 orders of magnitude in the biofilm matrix were considered as MBEC.

### Determination of F105 Cytotoxicity

Cytotoxicity of **F105** was determined using the CellTiter 96^®^ Aqueous Non-Radioactive Cell Proliferation Assay (Promega) using MCF-7 cells. The cells were cultured in DMEM – Dulbecco’s Modified Eagle’s Medium (Sigma–Aldrich) supplemented with 10% FBS, 2 mM L-glutamine, 100 mg/L penicillin and 100 mg/L streptomycin. Cells were seeded in 96-well plates with the density of 3000 cells per well and left overnight to allow for the attachment. Cells were next cultured at 37°C and 5% CO_2_ in the presence of **F105** at various concentrations from 1.25 to 160 mg/L. After 24 h of cultivation the cells were subjected to MTS-assay based on the cellular reduction of MTS (3-(4,5-dimethyl-2-yl)-5-(3-carboxymethoxyphenyl)-2-(4-sulfophenyl)-2*H*-tetrazolium) by the mitochondrial dehydroxygenase using phenazinemethosulfate (PMS) as the electron coupling reagent. The MTS tetrazolium compound was bioreduced by viable cells into a colored formazan product, which was measured on Tecan Infinite 200Pro at 550 nm. The concentration required to inhibit cellular dehydrogenase activity by 50% (CC_50_ value) was calculated as recommended by the manufacturer.

The mutagenicity of **F105** was evaluated in the Ames test with *Salmonella typhimurium* TA98, TA100 and TA102 strains as described in McCann and Ames (1976). The tested compound was considered to be mutagenic if the number of revertant colonies in the experiment was more than two times higher than that in the negative control (DMSO) and increased at higher **F105** concentrations (OECD, 1997).

### Data Analysis

All experiments were performed in biological triplicates with three repeats in each run. The data were analyzed and graphically visualized using GraphPad Prism version 6.00 for Windows (GraphPad Software, United States, www.graphpad.com). In each experiment, comparison against negative control has been performed using the non-parametric Kruskal–Wallis one-way analysis of variance test. Significant differences against respective controls were considered at *p* < 0.05 and are specified in the corresponding figure captions. Additionally, statistical significance of time-kill curves and dose-response curves have been assessed by linear regression analysis applied in log scales where appropriate. For the regression analysis data, shown confidence intervals also correspond to *p* < 0.05.

## Results

### Synthesis of Furanone F105

**FIGURE 1:**
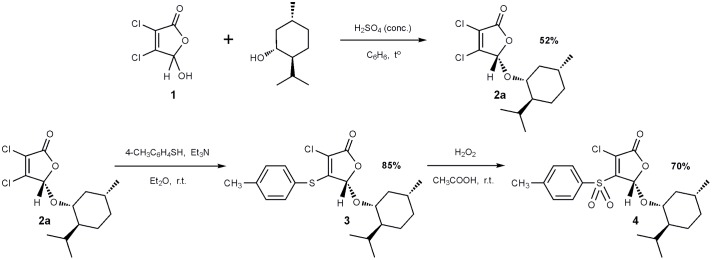
Synthesis of 2(5*H*)-furanone derivative **F105**.

The 2(5*H*)-furanone derivative **F105** **(4)** was synthesized in three steps from commercially available mucochloric acid **1** (see **Figure 1**). In the first stage, a mixture of diastereomers **2a** + **2b** was obtained in the reaction of mucochloric acid **1** with *l*-menthol in the presence of catalytic amounts of concentrated sulfuric acid as described previously (Fenske and Merzweiler, 1989). The pure stereoisomer **2a** with *S*-configuration of the carbon atom C^5^ of the γ-lactone ring was isolated in 52% yield after two recrystallizations from hexane (**Figure 1**).

Next a thiolation reaction of the isolated stereoisomer **2a** under basic catalysis was carried out. It is well-known that in the presence of triethylamine reactions of 5-alkoxy-2(5*H*)-furanones with thiols proceed with the regioselective substitution of the chlorine atom in the fourth position of the lactone ring (Kurbangalieva et al., 2007; Latypova et al., 2014). The reaction was performed in diethyl ether at room temperature with the equimolar ratio of furanone **2a**, *p*-thiocresol and triethylamine resulting in the novel optically pure thioether **3** with 85% yield. Thioether **3** was further subjected to oxidation to the corresponding sulfone **4** using a recently developed selective method (Latypova et al., 2014). The compound **3** was treated with 10-fold excess of 33% hydrogen peroxide in acetic acid at room temperature and the novel optically pure sulfonyl derivative of 2(5*H*)-furanone **4** (studied compound **F105**, **Figure 1**) was isolated in the form of colorless crystals in 70% yield. The structure of compounds **2**–**4** was characterized in detail by IR and NMR spectroscopy (Supplementary Figures S1–S5).

### Antimicrobial Activity of F105 on Planktonic *S. aureus*

**FIGURE 2:**
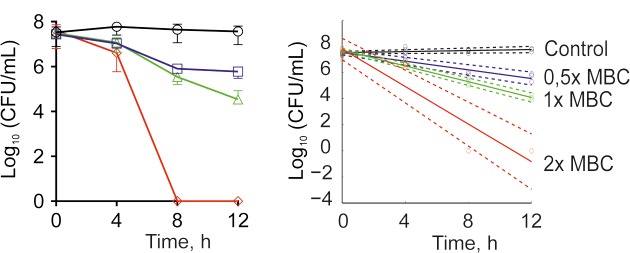
Time-kill curves of **F105** against MSSA cells. The numbers of CFU/mL were calculated by using drop plate approach **(A)** and respective dose-response curves were plotted providing residual CFU/mL as a function of exposition time **(B)**. **F105** is presented in different concentrations: untreated cells (black); 0.5× MBC (20 mg/L, blue); 1× MBC (40 mg/L, green); 2× MBC (80 mg/L, red). Full lines denote regression lines, while dashed lines denote corresponding 95% confidence intervals.

The antimicrobial properties of **F105** were determined on both methycillin-susceptible (MSSA) and -resistant (MRSA) *S. aureus* strains ATCC29213 and ATCC43300, respectively. The minimal inhibitory concentration (MIC) of **F105** for MSSA was found to be 10 mg/L (25 μM), and 20 mg/L (50 μM) for MRSA (see **Table 1**). The minimal bactericidal concentration (MBC) value of **F105** was found to be 40 mg/L in MSSA and 80 mg/L in MRSA. The time-kill curves revealed that all cells of MSSA exposed to **F105** at concentration of 2× MBC were killed within 8 h of treatment (**Figure 2A**). Alternatively, 1× MBC of **F105** led to the reduction in the number of viable cells by three orders of magnitude within 12 h. The dose-response curves (**Figure 2B**) confirm the concentration-dependence of **F105** effect on cells viability explicitly suggesting that **F105** exhibits biocidal activity. Interestingly, our earlier results indicate that the analogs of **F105** lacking either sulfonyl group (compound **3** on the **Figure 1**) or *l*-menthol moiety (**F70**, 3-chloro-5-hydroxy-4-[(4-methylphenylsulfonyl)]-2(5*H*)-furanone) exhibited no activity against *S. aureus* (Latypova et al., 2014; Kayumov et al., 2015a) (data not shown) suggesting the requirement of both functional groups for bactericidal activity, while in other works the presence of either sulfonyl group or *l*-menthol moiety increased antibacterial effects (Meadows and Gervay-Hague, 2006; Kudryavtsev et al., 2009; Rogers et al., 2010; Low et al., 2011).

We additionally assessed both MIC and MBC in 10 clinical isolates exhibiting a MRSA phenotype. Both values ranged within the MIC and MBC found in the MSSA laboratory standard strain with all MICs at 10 mg/L, and MBC at 20 mg/L (*n* = 5) or 40 mg/L (*n* = 5) (**Table 1**).

### Synergistic Effects of F105 with Other Antimicrobials on *S. aureus* Planktonic Cells

The synergism of **F105** in combination with various antibiotics on the bacterial growth was analyzed by the checkerboard assay (**Table 2** and Supplementary Table S1). Thereby synergism of **F105** was observed when combined with aminoglycosides. Particularly, the FICI values for **F105** were determined to be 0.33 ± 0.04 in combination with amikacin, 0.33 ± 0.16 with gentamicin and 0.44 ± 0.17 with kanamycin. Besides aminoglycosides, strong synergy has been observed also for benzalkonium chloride with FICI of 0.29 ± 0.09. Indifferent effects, but with relatively low FICI (between 0.51 and 0.75) indicating enhancement of antimicrobial activity were observed for **F105** in combination with daptomycin, rifampicin, ciprofloxacin, tetracycline, and linezolid; whereas the only additive effects were observed for combinations of **F105** with erythromycin and vancomycin. For deeper analysis of **F105** synergy with antimicrobials, the combined MICs of the antibiotic/**F105**-mixtures were plotted (as isoboles) and the effective concentrations of **F105** (EC_50_) leading to the twofold reduction of antibiotic’s MIC were calculated (**Table 2** and Supplementary Figure S6). The EC_50_ values of **F105** in combinations with amikacin, gentamycin, and kanamycin were determined to be 0.7, 0.7, and 1.3 mg/L, respectively. Only 0.5 mg/L of **F105** was required to reduce the MIC of benzalkonium chloride twofold. For other studied antibiotics EC_50_ values of **F105** were in the range of 4.3 and 9.5 mg/L.

**Table 2:** MIC and ECOFF values of various antibiotics against MSSA, FICI values of those antibiotics combined with **F105** and EC_50_ of **F105** reducing twice the MIC of the appropriate antibiotic.

	MIC^a^	ECOFF^a^	FICI_min_	EC_50_^a^
Benzalkonium chloride	1.00	ND	0.29 ± 0.09^b^	0.5
Amikacin	1.00	8.00	0.33 ± 0.04	0.7
Gentamicin	1.00	2.00	0.33 ± 0.16	0.7
Kanamycin	2.00	8.00	0.44 ± 0.17	1.3
Tetracycline	0.50	1.00	0.65 ± 0.10	4.3
Erythromycin	1.00	1.00	0.79 ± 0.19	4.9
Ciprofloxacin	0.50	1.00	0.68 ± 0.27	5.3
Ampicillin	0.25	ND	0.78 ± 0.23	7.1
Rifampicin	0.03	0.03	0.55 ± 0.02	8.3
Daptomycin	4.00	1.00	0.51 ± 0.01	9.5
Linezoild	4.00	4.00	0.55 ± 0.01	7.5
Vancomycin	2.00	2.00	0.92 ± 0.14	7.9


*^a^The concentrations are given in mg/L; ^b^FICI is given by the mean ± SD.*

### Anti-biofilm Activity of F105

**FIGURE 3:**
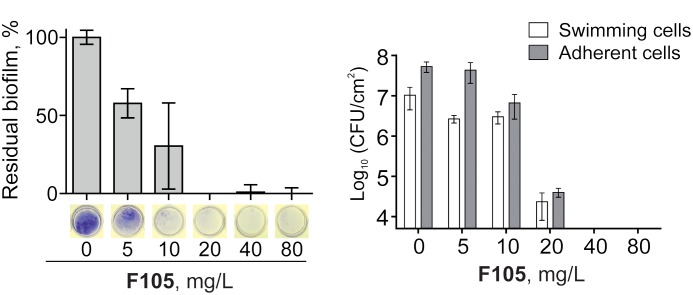
Effect of **F105** on the biofilm formation by MSSA. Cells were incubated for 24 h in the presence of different concentrations of **F105**, the biofilms were quantified after crystal-violet staining **(A)** and CFU counting **(B)**; thereby the crystal-violet method correlates with the biomass and CFU/mL with viable cells.

**FIGURE 4:**
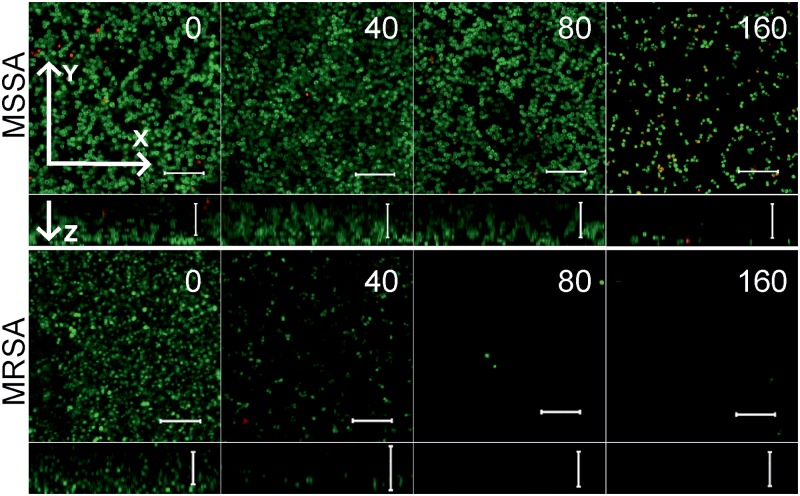
The biofilm-preventing activity (BPC) of **F105** against MSSA **(upper)** and MRSA **(lower)** analyzed by CLSM. **F105** was added prior the inoculation with following cell grow for 24 h. The images show a plan view on a basal biofilm layer (indicated by X and Y axis) and a cross section thought the biofilm (Z axis). The scale bars indicate 10 μm.

Since the anti-biofilm activity of furanones was reported for some compounds (Ren et al., 2001; Hentzer et al., 2002; Lonn-Stensrud et al., 2009; Fedorova et al., 2013), the biofilm preventing concentration (BPC) of **F105** for the MSSA laboratory standard strain was assessed first by two different methods. The crystal violet staining method revealed that **F105** completely inhibited the biofilm formation of the MSSA at the concentration of 20 mg/L (**Figure 3A**); whereas, applying direct counting of viable cell in the biofilm the BPC was found to be 40 mg/L (**Figure 3B** and **Table 1**). Since the amount of viable planktonic cells (swimming cells) also decreased by three orders of magnitude at 20 mg/L of **F105**, we suggested that the biofilm suppression was rather the consequence of cell growth repression. The CLSM analysis confirmed the BPC of **F105** of 40 mg/L for MSSA (**Figure 4**), thus we estimated that these direct methods are more adequate compared to the indirect crystal violet staining. The determined BPC of MRSA was 80 mg/L according to the CFU/ml counting and 40 mg/L according to the CLSM (**Table 1** and **Figure 4**). The BPC was measured by the drop plate method in the clinical MRSA isolates resulting in 20 mg/L in nine and 40 mg/L in one isolate (**Table 1**).

**FIGURE 5:**
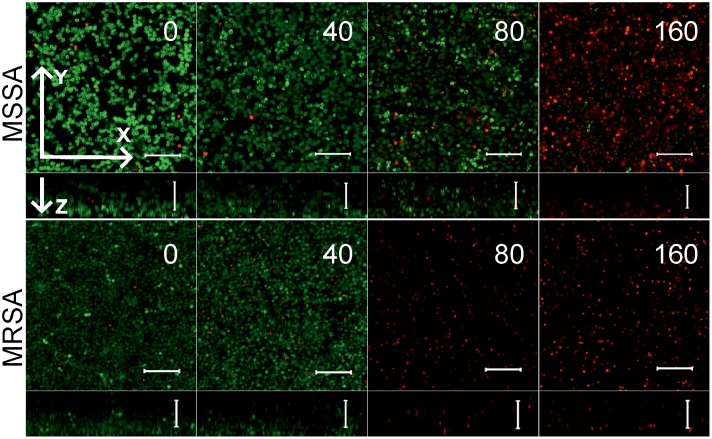
The antimicrobial activity of **F105** against biofilm-embedded MSSA **(upper)** and MRSA **(lower)** analyzed by CLSM. **F105** was added to established 24 h old biofilms with following 24 h cultivation. The scale bars indicate 10 μm.

**FIGURE 6:**
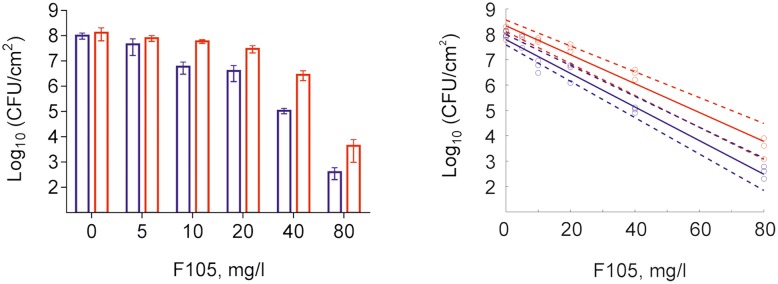
Effect of **F105** on MSSA cells viability in culture liquid (blue) and within the 24 h old biofilm layer (red) **(A)** and corresponding dose-response curves **(B)** estimated by linear regression in the logarithmic scale (full lines) with 95% confidence intervals for the regression coefficients (dashed lines) of swimming (planctonic and detached) and adherent cells. **F105** was added to 24 h old biofilms with additional 24 h cultivation. Significant differences with control could be observed at concentrations from 10 mg/L and above for swimming cells as well as from 20 mg/L and above for adherent cells by Kruskal–Wallis test at *p* < 0.05.

To further analyze, whether **F105** not only inhibits the biofilm formation but also exhibits activity against already established biofilms, 24 h-old *S. aureus* biofilms of the MSSA and MRSA laboratory standard strains were treated with different concentrations of **F105**. The CLSM analysis of the MBEC indicated that the treatment with **F105** did not lead to any visibly remarkable decrease of the biofilm thickness (**Figure 5**), while the ratio of dead/viable cell increased significantly in the concentration dependent manner. Biofilm-embedded cells of MRSA were nearly completely killed at 80 mg/L of **F105**, whereas MSSA cells were killed at 160 mg/L. However, the CFU/mL of the planktonic cells and the adherent cells of MSSA biofilm cultures revealed a reduction in the number of viable cells by three orders of magnitude at 80 mg/L of **F105** for both attached and detached cells (**Figure 6**) indicating that CLSM results vary depending on the screened section and the CFU/mL values are more reliable.

**FIGURE 7:**
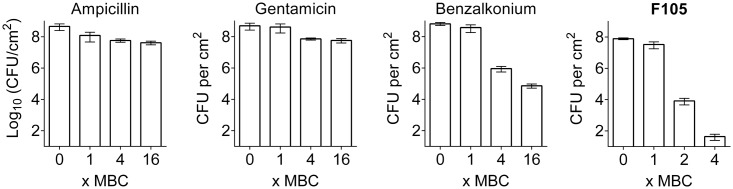
The comparison of antimicrobial activity of **F105** and various antibiotics against biofilm embedded MSSA cells. Antimicrobials were added in wells with established 24 h old biofilms and incubation was continued for 24 h at 35°C. The concentrations are given in relative units as X-fold MBC, which were as follows: 8 mg/L for ampicillin, 4 mg/L for gentamycin, 2 mg/L for benzalkonium, and 40 mg/L for **F105**.

To compare the biofilm activity of **F105** against other antimicrobials, similar experiments were performed with ampicillin, gentamicin, and benzalkonium chloride (see Supplementary Table S2 for MBC values). A fourfold MBC of benzalkonium chloride was required to reduce the number of viable biofilm-embedded cells by three orders of magnitude, while even 16-fold MBC of gentamicin or ampicillin led to only 10-fold decrease of viable cells in the biofilm (**Figure 7**). In marked contrast, twofold MBC of **F105** reduced the viable cells of the MSSA biofilm by more than four orders of magnitude (**Figure 7** and Supplementary Figure S7).

### Cytotoxicity and Mutagenicity

No mutagenicity of **F105** was detected in the Ames test using *Salmonella typhimurium* TA98, TA100, and TA102 strains at the furanone concentrations up to 80 mg/L without (Supplementary Table S3) and with (Supplementary Table S4) metabolic activation. The cytotoxicity test provided with CC_50_ values of 40.3 mg/L for MCF-7 cells (a human breast adenocarcinoma cell line) but only 8.1 mg/L for skin fibroblast suggesting that the current structure of **F105** might be too toxic for direct use for systemic therapeutic in humans and requires further modification of the structure in order to reduce its toxicity and improve its solubility.

## Discussion

The biofilm formation by the methicillin-resistant *S. aureus* cells on wounds and surfaces contacting with different tissues makes bacteria inaccessible to both antimicrobials and the immune system of the host. The discovery of the natural furanones derivatives exhibiting biofilm suppression activity (Ren et al., 2001, 2004) gave the rise to investigations of these compounds as anti-biofilm agents (Brackman and Coenye, 2015). While many 2(5*H*)-furanone derivatives interfere with AI-II quorum-sensing systems of Gram-negative bacteria blocking the biofilm formation (Ren et al., 2001; Hentzer et al., 2003), a number of furanones were shown to be active against gram-positive *S. epidermidis* and *B. subtilis* (Heck and Stuetz, 1988; Ren et al., 2004; Lonn-Stensrud et al., 2009; Kayumov et al., 2015a; Trizna et al., 2015).

Here, we have shown that 3-chloro-5(*S*)-[(1*R*,2*S*,5*R*)-2-isopropyl-5-methylcyclohexyloxy]-4-[4-methylphenylsulfonyl]-2(5*H*)-furanone (**F105**), carrying sulfonyl and *l*-menthol moieties, exhibits antibacterial activity against both planktonic and biofilm-embedded *S. aureus*. There were some reasons, why we have chosen these modifications to improve the antimicrobial activity of this furanone derivative. In particular, menthol has been shown previously to exhibit biofilm inhibiting and biofilm eliminating effects (Kifer et al., 2016) and it is one of the most effective terpenes used to enhance the dermal penetration of pharmaceuticals (Kamatou et al., 2013). Moreover, introduction of the *l*-menthol moiety into carbamate derivatives significantly increases their anti-biofilm properties toward MRSA (Rogers et al., 2010). It could be also shown that *l*-menthol increased CH_2_ stretching frequencies on either side of lipid transition and lowed the Tm by ∼2–8°C suggesting that *l*-menthol disrupts the interlamellar hydrogen-bonding network at the polar head group region and increases the hydration levels of the model lipid system, most probably by forming new aqueous channels (Narishetty and Panchagnula, 2005). Therefore, it could be speculated that *l*-menthol moiety acts similarly in the biofilm and facilitates the diffusion of **F105** into the biofilm matrix and probably through the cell membrane. Several researchers reported that sulfonyl-containing compounds effectively repressed the growth of *Staphylococci* (Meadows and Gervay-Hague, 2006; Kudryavtsev et al., 2009; Low et al., 2011) suggesting antibacterial activity of the sulfonyl moiety (Mohan et al., 2014; Thirukovela et al., 2017). In contrast, the analogs of **F105** lacking either sulfonyl group (compound **3** on the **Figure 1**) or *l*-menthol moiety (3-chloro-5-hydroxy-4-[(4-methylphenylsulfonyl)]-2(5*H*)-furanone) exhibited no activity against *S. aureus* (Latypova et al., 2014; Kayumov et al., 2015a) suggesting the requirement of both functional groups for bactericidal activity of **F105**.

The MIC was twofold higher (20 mg/L) in the MRSA compared to the MSSA. This difference between the MSSA and MRSA laboratory strains might be attributed to the alternation of the peptidoglycan composition due to the methicillin resistance as described previously (de Jonge and Tomasz, 1993), which might hamper efficient cell penetration, while we did not investigate this hypothesis explicitly. The reduced susceptibility might be also related to the SCC*mec* cassette that harbor, besides the methicillin-resistance determinants (*mecA* allels), up to more than 50 open reading frames. While some of these ORFs are known to be responsible for various resistances (e.g., the *cadDX* or *arsRBC* and *arsDARBC* operons for cadmium and arsenate resistance) (Li et al., 2011) the other are of unknown functions and might also influence the susceptibility to **F105**. However, this has not been analyzed in this work and is currently only a hypothetical assumption. The analysis of clinical MRSA isolates also indicates that there might be in general variability in the MIC between the strains, because 9/10 clinical MRSA isolates showed an MIC of **F105** of 10 mg/L. Thus we assume that 10–20 mg/L **F105** (corresponding to 25–50 μM) might be the effective MIC-range in many *staphylococci*. Comparing **F105** to other furanone derivatives, which MICs in different *S. aureus* strains were reported to range between 4.65 mg/L (15 μM) (Kuehl et al., 2009) and 16 mg/L (107 μM) (Lattmann et al., 2005), the antimicrobial activity of **F105** is moderate. However, further experiments in a broad range of clinical isolates will be needed to determine the MIC distributions of various furanones in the staphylococcal population to draw conclusion of the ecological cut-off (ECOFF), which separates the wild-type population from resistant isolate.

Comparing to other furanone derivatives and antimicrobials, the effect of **F105** in established biofilms was remarkably strong and exceeded the activity of conventional antibiotics by several orders of magnitude. This could be attributed to the assumption that, leaving the biofilm structure almost unchanged, **F105** easily penetrates the extracellular biofilm matrix and kills the matrix-embedded cells, in marked contrast to other antibiotics like gentamicin, ampicillin, chloramphenicol, or vancomycin (Parra-Ruiz et al., 2010; Singh et al., 2010; Kayumov et al., 2015b; Trizna et al., 2016). There are only few antibiotics with specific mechanisms of action, which show some activity in *S. aureus* biofilms, e.g., rifampin and to a lesser extent daptomycin or fluoroquinolones (Garcia et al., 2013; Siala et al., 2014; Stein et al., 2016). Rifampin inhibits RNA translation, fluoroquinolones inhibit DNA transcription and amplification, while daptomycin perforates the bacterial cell membrane. The mechanism of **F105** action remains so far elusive. Our preliminary screening of the potential molecular targets of the **F105** by LC–MS mass spectrometry analysis indicated that the intracellular levels of many proteins in *S. aureus* either decreased or increased when growing at 0.5x MIC of **F105**. Most of those proteins are enzymes involved in different cellular metabolic processes (Supplementary Tables S5, S6), while it remains currently unclear, whether these processes are directly (by interaction with specific regulators) or indirectly (e.g., in course of general stress response) impacted by **F105**. These data provide no evidence that **F105** targets quorum sensing-depending processes and thus it remains rather unlikely that the observed intra-biofilm killing is conferred by quorum sensing inhibition alone. We also cannot explain the opposite biofilm-killing efficacy of **F015** on both MRSA and MSSA laboratory strains comparing to the MIC and MBC. We can only hypothesize that this effect might also originate from intra-species variability and differentially expressed **F105** targets.

No mutagenicity of **F105** was detected in the Ames test using *Salmonella typhimurium* TA98, TA100 and TA102 strains at the furanone concentrations up to 80 mg/L without (Supplementary Table S3) and with (Supplementary Table S4) metabolic activation. The cytotoxicity test provided with CC_50_ values of 40.3 mg/L for MCF-7 cells (a human breast adenocarcinoma cell line) but only 8.1 mg/L for the skin fibroblast suggesting that the current structure of **F105** might be too toxic for direct use in systemic therapeutics in humans and requires further modification of the structure in order to lower its toxicity and improve its solubility. The nearest derivative of **F105** carrying arylsulfanyl substituent instead of arylsulfonyl group (compound **3** from the scheme 1) or lacking the *l*-menthol moiety (sulfone **F70;** Kayumov et al., 2015a) demonstrated higher CC_50_ values (32 and 45 mg/L, respectively), but exhibited no antibacterial activity even at concentrations up to 256 mg/L. These data suggest that the cytotoxicity of **F105** presumably originates from the chlorinated 2(5*H*)-furanone fragment (Ren et al., 2004; Kitty et al., 2015) rather than from either sulfonyl or *l*-menthol moieties which are responsible for antibacterial effects of the studied compound.

Since the number of patients with skin wounds with MRSA infections and MRSA-related hospitalizations and deaths are continually increasing (Kalita et al., 2015), the synergy of **F105** with aminoglycosides and benzalkonium chloride makes it an attractive starting point for the development of therapeutic strategies for the skin wounds treatment. Furthermore, some biocides with relatively high cytotoxicity are widely used in practice as disinfectants or local antiseptics, with miramistin or benzalkonium chloride, two biocides belonging to quaternary ammonium salts being a prominent example. Both are cytotoxic in direct contact with typical human cells, but the compounds are known as effective antiseptics for the local treatment of infected wounds with low side effects (Bernstein, 2000; Fromm-Dornieden et al., 2015). In particular, the CC_50_ value of benzalkonium chloride for the normal human fibroblasts was reported to be 6.7 mg/L, with CC_50_/MBC ratio of 0.05 (Damour et al., 1992). For **F105** the CC_50_/MBC ratio was found to be 0.2, suggesting its higher or comparable therapeutic index in comparison with benzalkonium chloride, which is widely used as a biocide for outer treatment (Akimitsu et al., 1999; Bernstein, 2000). Taking in account that already 0.5–0.7 mg/L of **F105** (∼15-fold less than CC_50_) decreases the MICs of aminoglycosides and benzalkonium chloride twofold, and the ability of **F105** to target the biofilm-embedded *Staphylococci*, its chemotype looks as attractive tool for combination with antimicrobials to reduce their therapeutic concentrations, as well as to decrease their side effects and to enhance the efficacy of treatment of both planktonic and biofilm-embedded bacteria.

## Author Contributions

IS, ET, DB, RS, LL, and ER performed the experiments. AKa, AKu, MK-S, RF, and OM conducted the experiments. IS, MP, MB, AKa, AKu, and OM analyzed the results. IS, AKa, AKu, and OM prepared figures and graphs and wrote the manuscript. AKa, AKu, MB, OM, and MP revised the manuscript. All the authors read and approved the final version of the manuscript.

## Conflict of Interest Statement

The authors declare that the research was conducted in the absence of any commercial or financial relationships that could be construed as a potential conflict of interest.

## References

[B1] Akimitsu N. Hamamoto H. Inoue R. Shoji M. Akamine A. Takemori K. (1999). Increase in resistance of methicillin-resistant *Staphylococcus aureus* to beta-lactams caused by mutations conferring resistance to benzalkonium chloride, a disinfectant widely used in hospitals. *Antimicrob. Agents Chemother.* 43 3042–3043. 10651623 10.1128/aac.43.12.3042 PMC89614

[B2] Atshan S. S. Shamsudin M. N. Sekawi Z. Lung L. T. T. Barantalab F. Liew Y. K. (2015). Comparative proteomic analysis of extracellular proteins expressed by various clonal types of *Staphylococcus aureus* and during planktonic growth and biofilm development. *Front. Microbiol.* 6:524. 10.3389/fmicb.2015.00524 26089817 PMC4454047

[B3] Baidamshina D. R. Trizna E. Y. Holyavka M. G. Bogachev M. I. Artyukhov V. G. Akhatova F. S. (2017). Targeting microbial biofilms using Ficin, a nonspecific plant protease. *Sci. Rep.* 7:46068. 10.1038/srep46068 28387349 PMC5384253

[B4] Bassetti M. Carnelutti A. Righi E. (2017). The role of methicillin-resistant *Staphylococcus aureus* in skin and soft tissue infections. *Curr. Opin. Infect. Dis.* 30 150–157. 10.1097/qco.0000000000000353 28079631

[B5] Bernstein I. L. (2000). Is the use of benzalkonium chloride as a preservative for nasal formulations a safety concern? A cautionary note based on compromised mucociliary transport. *J. Allergy Clin. Immunol.* 105 39–44. 10.1016/s0091-6749(00)90175-1 10629450

[B6] Bortolin M. Bidossi A. De Vecchi E. Avveniente M. Drago L. (2017). In vitro antimicrobial activity of chlorquinaldol against microorganisms responsible for skin and soft tissue infections: comparative evaluation with gentamicin and fusidic acid. *Front. Microbiol.* 8:10. 10.3389/fmicb.2017.01039 28642751 PMC5462991

[B7] Brackman G. Coenye T. (2015). Quorum sensing inhibitors as anti-biofilm agents. *Curr. Pharm. Des.* 21 5–11. 10.2174/1381612820666140905114627 25189863

[B8] Bremer F. Grade S. Kohorst P. Stiesch M. (2011). In vivo biofilm formation on different dental ceramics. *Quintessence Int.* 42 565–574. 21716984

[B9] Conlon B. P. (2014). *Staphylococcus aureus* chronic and relapsing infections: evidence of a role for persister cells an investigation of persister cells, their formation and their role in *S. aureus* disease. *Bioessays* 36 991–996. 10.1002/bies.201400080 25100240

[B10] Cosgrove S. E. Fowler V. G. Jr. (2008). Management of methicillin-resistant *Staphylococcus aureus* bacteremia. *Clin. Infect. Dis.* 46 S386–S393. 10.1086/533595 18462094

[B11] Cosgrove S. E. Kaye K. S. Eliopoulous G. M. Carmeli Y. (2002). Health and economic outcomes of the emergence of third-generation cephalosporin resistance in *Enterobacter* species. *Arch. Intern. Med.* 162 185–190. 10.1001/archinte.162.2.185 11802752

[B12] Damour O. Hua S. Z. Lasne F. Villain M. Rousselle P. Collombel C. (1992). Cytotoxicity evaluation of antibiotics on cultured human fibroblasts ans kreatinocytes. *Burns* 18 479–485. 10.1016/0305-4179(92)90180-3 1489497

[B13] de Jonge B. L. Tomasz A. (1993). Abnormal peptidoglycan produced in a methicillin-resistant strain of *Staphylococcus aureus* grown in the presence of methicillin: functional role for penicillin-binding protein 2A in cell wall synthesis. *Antimicrob. Agents Chemother.* 37 342–346. 10.1128/AAC.37.2.342 8452368 PMC187665

[B14] den Hollander J. G. Mouton J. W. Verbrugh H. A. (1998). Use of pharmacodynamic parameters to predict efficacy of combination therapy by using fractional inhibitory concentration kinetics. *Antimicrob. Agents Chemother.* 42 744–748. 9559776 10.1128/aac.42.4.744 PMC105535

[B15] Doll K. Jongsthaphongpun K. L. Stumpp N. S. Winkel A. Stiesch M. (2016). Quantifying implant-associated biofilms: comparison of microscopic, microbiologic and biochemical methods. *J. Microbiol. Methods* 130 61–68. 10.1016/j.mimet.2016.07.016 27444546

[B16] Donlan R. M. (2002). Biofilms: microbial life on surfaces. *Emerg. Infect. Dis.* 8 881–890. 10.3201/eid0809.020063 12194761 PMC2732559

[B17] Donlan R. M. Costerton J. W. (2002). Biofilms: survival mechanisms of clinically relevant microorganisms. *Clin. Microbiol. Rev.* 15 167–193. 10.1128/cmr.15.2.167-193.2002 11932229 PMC118068

[B18] Eliopoulos G. Moellering R. C. Jr. (1996). “Antimicrobial combinations,” in *Antibiotics in Laboratory Medicine*, 4th Edn, ed. Lorian V. (Baltimore, MD: The Williams & Wilkins Co), 330–396.

[B19] European Committee for Antimicrobial Susceptibility Testing (EUCAST) of the European Society of Clinical Microbiology and Infectious Diseases (ESCMID). (2000). Terminology relating to methods for the determination of susceptibility of bacteria to antimicrobial agents. *Clin. Microbiol. Infect.* 6 503–508. 10.1046/j.1469-0691.2000.00149.x 11168186

[B20] Fagerlund A. Langsrud S. Heir E. Mikkelsen M. I. Moretro T. (2016). Biofilm matrix composition affects the susceptibility of food associated staphylococci to cleaning and disinfection agents. *Front. Microbiol.* 7:856. 10.3389/fmich.2010.00856 27375578 PMC4893552

[B21] Fedorova K. P. Scharafutdinov I. S. Turbina E. Y. Bogachev M. I. Ilinskaja O. N. Kayumov A. R. (2013). C-terminus of transcription factor TnrA from Bacillus subtilis controls DNA-binding domain activity but is not required for dimerization. *Mol. Biol.* 47 293–298. 10.1134/S0026893313020052 23808168

[B22] Fenske D. Merzweiler K. (1989). Synthesis of a new chiral phosphine ligand. *J. Chem. Sci.* 44 879–883.

[B23] Forrest G. N. Tamura K. (2010). Rifampin combination therapy for nonmycobacterial infections. *Clin. Microbiol. Rev.* 23 14–34. 10.1128/cmr.00034-09 20065324 PMC2806656

[B24] Fromm-Dornieden C. Rembe J. D. Schafer N. Bohm J. Stuermer E. K. (2015). Cetylpyridinium chloride and miramistin as antiseptic substances in chronic wound management - prospects and limitations. *J. Med. Microbiol.* 64 407–414. 10.1099/jmm.0.000034 25681322

[B25] Garcia L. G. Lemaire S. Kahl B. C. Becker K. Proctor R. A. Denis O. (2013). Antibiotic activity against small-colony variants of *Staphylococcus aureus*: review of in vitro, animal and clinical data. *J. Antimicrob. Chemother.* 68 1455–1464. 10.1093/jac/dkt072 23485724

[B26] Heck R. Stuetz A. (1988). Antimycotic-6-phenyl-2-hexen-4-ynamines. U.S. Patent No. EP 0254677 A1.

[B27] Hentzer M. Riedel K. Rasmussen T. B. Heydorn A. Andersen J. B. Parsek M. R. (2002). Inhibition of quorum sensing in *Pseudomonas aeruginosa* biofilm bacteria by a halogenated furanone compound. *Microbiology* 148 87–102. 10.1099/00221287-148-1-87 11782502

[B28] Hentzer M. Wu H. Andersen J. B. Riedel K. Rasmussen T. B. Bagge N. (2003). Attenuation of *Pseudomonas aeruginosa* virulence by quorum sensing inhibitors. *EMBO J.* 22 3803–3815. 10.1093/emboj/cdg366 12881415 PMC169039

[B29] Herigstad B. Hamilton M. Heersink J. (2001). How to optimize the drop plate method for enumerating bacteria. *J. Microbiol. Methods* 44 121–129. 10.1016/s0167-7012(00)00241-4 11165341

[B30] Hodille E. Rose W. Diep B. A. Goutelle S. Lina G. Dumitrescu O. (2017). The role of antibiotics in modulating virulence in *Staphylococcus aureus*. *Clin. Microbiol. Rev.* 30 887–917. 10.1128/CMR.00120-16 28724662 PMC5608880

[B31] Kali A. Bhuvaneshwar D. Charles P. M. Seetha K. S. (2016). Antibacterial synergy of curcumin with antibiotics against biofilm producing clinical bacterial isolates. *J. Basic Clin. Pharm.* 7 93–96. 10.4103/0976-0105.183265 27330262 PMC4910474

[B32] Kalita S. Devi B. Kandimalla R. Sharma K. K. Sharma A. Kalita K. (2015). Chloramphenicol encapsulated in poly-epsilon-caprolactone-pluronic composite: nanoparticles for treatment of MRSA-infected burn wounds. *Int. J. Nanomed.* 10 2971–2984. 10.2147/ijn.s75023 25931822 PMC4404939

[B33] Kamatou G. P. Vermaak I. Viljoen A. M. Lawrence B. M. (2013). Menthol: a simple monoterpene with remarkable biological properties. *Phytochemistry* 96 15–25. 10.1016/j.phytochem.2013.08.005 24054028

[B34] Kayumov A. R. Khakimullina E. N. Sharafutdinov I. S. Trizna E. Y. Latypova L. Z. Hoang Thi L. (2015a). Inhibition of biofilm formation in *Bacillus subtilis* by new halogenated furanones. *J. Antibiotics* 68 297–301. 10.1038/ja.2014.143 25335695

[B35] Kayumov A. R. Nureeva A. A. Trizna E. Y. Gazizova G. R. Bogachev M. I. Shtyrlin N. V. (2015b). New derivatives of pyridoxine exhibit high antibacterial activity against biofilm-embedded staphylococcus cells. *Biomed Res. Int.* 2015:890968. 10.1155/2015/890968 26839888 PMC4709599

[B36] Kifer D. Muzinic V. Klaric M. S. (2016). Antimicrobial potency of single and combined mupirocin and monoterpenes, thymol, menthol and 1,8-cineole against *Staphylococcus aureus* planktonic and biofilm growth. *J. Antibiot.* 69 689–696. 10.1038/ja.2016.10 26883392

[B37] Kitty H. Samuel K. Daniel C. Renxun C. Mark W. Naresh K. (2015). “Development of fimbrolides, halogenated furanones and their derivatives as antimicrobial agents,” in *Antibacterial Surfaces*, ed. Elena Ivanova R. C. (Cham: Springer International Publishing), 149–170.

[B38] Klinger-Strobel M. Suesse H. Fischer D. Pletz M. W. Makarewicz O. (2016). A novel computerized cell count algorithm for biofilm analysis. *PLOS ONE* 11:e0154937. 10.1371/journal.pone.0154937 27149069 PMC4858220

[B39] Kudryavtsev K. V. Bentley M. L. McCafferty D. G. (2009). Probing of the cis-5-phenyl proline scaffold as a platform for the synthesis of mechanism-based inhibitors of the *Staphylococcus aureus* sortase SrtA isoform. *Bioorg. Med. Chem.* 17 2886–2893. 10.1016/j.bmc.2009.02.008 19269184 PMC2663005

[B40] Kuehl R. Al-Bataineh S. Gordon O. Luginbuehl R. Otto M. Textor M. (2009). Furanone at subinhibitory concentrations enhances staphylococcal biofilm formation by luxS repression. *Antimicrob. Agents Chemother.* 53 4159–4166. 10.1128/aac.01704-08 19620329 PMC2764226

[B41] Kurbangalieva A. R. Devyatova N. F. Bogdanov A. V. Berdnikov E. A. Mannafov T. G. Krivolapov D. B. (2007). Synthesis of novel arylthio derivatives of mucochloric acid. *Phosphorus Sulfur Silicon Relat. Elem.* 182 607–630. 10.1080/10426500601015989

[B42] Lattmann E. Dunn S. Niamsanit S. Sattayasai N. (2005). Synthesis and antibacterial activities of 5-hydroxy-4-amino-2(5*H*)-furanones. *Bioorg. Med. Chem. Lett.* 15 919–921. 10.1016/j.bmcl.2004.12.051 15686887

[B43] Latypova L. Z. Saigitbatalova E. S. Chulakova D. R. Lodochnikova O. A. Kurbangalieva A. R. Berdnikov E. A. (2014). Sulfides, sulfones, and sulfoxides of the furan-2(5*H*)-one series. synthesis and structure. *Russ. J. Organ. Chem.* 50 521–534. 10.1134/s1070428014040149

[B44] Leclercq R. Canton R. Brown D. F. Giske C. G. Heisig P. MacGowan A. P. (2013). EUCAST expert rules in antimicrobial susceptibility testing. *Clin. Microbiol. Infect* 19 141–160. 10.1111/j.1469-0691.2011.03703.x 22117544

[B45] Lewis K. (2001). Riddle of biofilm resistance. *Antimicrob. Agents Chemother.* 45 999–1007. 10.1128/AAC.45.4.999-1007.2001 11257008 PMC90417

[B46] Li S. S. Skov R. L. Han X. Larsen A. R. Larsen J. Sorum M. (2011). Novel types of staphylococcal cassette chromosome mec elements identified in clonal complex 398 methicillin-resistant *Staphylococcus aureus* strains. *Antimicrob. Agents Chemother.* 55 3046–3050. 10.1128/aac.01475-10 21422209 PMC3101438

[B47] Lonn-Stensrud J. Landin M. A. Benneche T. Petersen F. C. Scheie A. A. (2009). Furanones, potential agents for preventing *Staphylococcus epidermidis* biofilm infections? *J. Antimicrob. Chemother.* 63 309–316. 10.1093/jac/dkn501 19098295

[B48] Low E. Kim B. Francavilla C. Shiau T. P. Turtle E. D. O’Mahony D. J. R. (2011). Structure stability/activity relationships of sulfone stabilized N,N-dichloroamines. *Bioorg. Med. Chem. Lett.* 21 3682–3685. 10.1016/j.bmcl.2011.04.084 21570284

[B49] McCann J. Ames B. N. (1976). A simple method for detecting environmental carcinogens as mutagens. *Ann. N. Y. Acad. Sci.* 271 5–13. 10.1111/j.1749-6632.1976.tb23086.x 793475

[B50] McCarthy H. Rudkin J. K. Black N. S. Gallagher L. O’Neill E. O’Gara J. P. (2015). Methicillin resistance and the biofilm phenotype in *Staphylococcus aureus*. *Front. Cell. Infect. Microbiol.* 5:1 10.3389/fcimb.2015.00001 PMC4309206 25674541

[B51] Meadows D. C. Gervay-Hague J. (2006). Vinyl sulfones: synthetic preparations and medicinal chemistry applications. *Med. Res. Rev.* 26 793–814. 10.1002/med.20074 16788979

[B52] Merritt J. H. Kadouri D. E. O’Toole G. A. (2005). Growing and analyzing static biofilms. *Curr. Protoc. Microbiol. Chapter* 1 Unit 1B. 1. 10.1002/9780471729259.mc01b01s00 18770545 PMC4568995

[B53] Mohan N. R. Sreenivasa S. Manojkumar K. E. Rao T. M. C. Thippeswamy B. S. Suchetan P. A. (2014). Synthesis, antibacterial, anthelmintic and anti-inflammatory studies of novel methylpyrimidine sulfonyl piperazine derivatives. *J. Braz. Chem. Soc.* 25 1012–1020. 10.5935/0103-5053.20140073

[B54] Naicker P. R. Karayem K. Hoek K. G. P. Harvey J. Wasserman E. (2016). Biofilm formation in invasive *Staphylococcus aureus* isolates is associated with the clonal lineage. *Microb. Pathog.* 90 41–49. 10.1016/j.micpath.2015.10.023 26546719

[B55] Narishetty S. T. Panchagnula R. (2005). Effect of L-menthol and 1,8-cineole on phase behavior and molecular organization of SC lipids and skin permeation of zidovudine. *J. Control. Release* 102 59–70. 10.1016/j.jconrel.2004.09.016 15653134

[B56] Odds F. C. (2003). Synergy, antagonism, and what the chequerboard puts between them. *J. Antimicrob. Chemother.* 52:1. 10.1093/jac/dkg301 12805255

[B57] OECD (1997). *Test No. 471: Bacterial Reverse Mutation Test*. Paris: OECD.

[B58] Parra-Ruiz J. Vidaillac C. Rose W. E. Rybak M. J. (2010). Activities of high-dose daptomycin, vancomycin, and moxifloxacin alone or in combination with clarithromycin or rifampin in a novel in vitro model of *Staphylococcus aureus* biofilm. *Antimicrob. Agents Chemother.* 54 4329–4334. 10.1128/aac.00455-10 20696880 PMC2944618

[B59] Peeters E. Nelis H. J. Coenye T. (2008). Comparison of multiple methods for quantification of microbial biofilms grown in microtiter plates. *J. Microbiol. Methods* 72 157–165. 10.1016/j.mimet.2007.11.010 18155789

[B60] Percival S. L. Finnegan S. Donelli G. Vuotto C. Rimmer S. Lipsky B. A. (2016). Antiseptics for treating infected wounds: efficacy on biofilms and effect of pH. *Crit. Rev. Microbiol.* 42 293–309. 10.3109/1040841x.2014.940495 25159044

[B61] Ren D. C. Bedzyk L. A. Setlow P. England D. F. Kjelleberg S. Thomas S. M. (2004). Differential gene expression to investigate the effect of (5Z)-4-bromo-5-(bromomethylene)-3-butyl-2(5*H*)-furanone on *Bacillus subtilis*. *Appl. Environ. Microbiol.* 70 4941–4949. 10.1128/aem.70.8.4941-4949.2004 15294834 PMC492336

[B62] Ren D. C. Sims J. J. Wood T. K. (2001). Inhibition of biofilm formation and swarming of *Escherichia coli* by (5Z)-4-bromo-5(bromomethylene)-3-butyl-2(5*H*)-furanone. *Environ. Microbiol.* 3 731–736. 10.1046/j.1462-2920.2001.00249.x. 11846763

[B63] Rogers S. A. Whitehead D. C. Mullikin T. Melander C. (2010). Synthesis and bacterial biofilm inhibition studies of ethyl N-(2-phenethyl) carbamate derivatives. *Organ. Biomol. Chem.* 8 3857–3859. 10.1039/c0ob00063a 20617245

[B64] Roy R. Tiwari M. Donelli G. Tiwari V. (2017). Strategies for combating bacterial biofilms: a focus on anti-biofilm agents and their mechanisms of action. *Virulence* 10.1080/21505594.2017.1313372 [Epub ahead of print]. 28362216 PMC5955472

[B65] Sanchez-Vizuete P. Orgaz B. Aymerich S. Le Coq D. Briandet R. (2015). Pathogens protection against the action of disinfectants in multispecies biofilms. *Front. Microbiol.* 6:705. 10.3389/fmicb.2015.00705 26236291 PMC4500986

[B66] Sharafutdinov I. Shigapova Z. Baltin M. Akhmetov N. Bogachev M. Kayumov A. (2016). HtrA protease from *Bacillus subtilis* suppresses the bacterial fouling of the rat skin injuries. *Bionanoscience* 6 564–567. 10.1007/s12668-016-0281-2

[B67] Siala W. Mingeot-Leclercq M. P. Tulkens P. M. Hallin M. Denis O. Van Bambeke F. (2014). Comparison of the antibiotic activities of daptomycin, vancomycin, and the investigational fluoroquinolone delafloxacin against biofilms from *Staphylococcus aureus* clinical isolates. *Antimicrob. Agents Chemother.* 58 6385–6397. 10.1128/AAC.03482-14 25114142 PMC4249400

[B68] Singh R. Ray P. Das A. Sharma M. (2010). Penetration of antibiotics through *Staphylococcus aureus* and *Staphylococcus epidermidis* biofilms. *J. Antimicrob. Chemother.* 65 1955–1958. 10.1093/jac/dkq257 20615927

[B69] Stein C. Makarewicz O. Forstner C. Weis S. Hagel S. Loffler B. (2016). Should daptomycin-rifampin combinations for MSSA/MRSA isolates be avoided because of antagonism? *Infection* 44 499–504. 10.1007/s15010-016-0874-2 26797915

[B70] Thirukovela N. S. Kankala S. Kankala R. K. Paidakula S. Gangula M. R. Vasam C. S. (2017). Regioselective synthesis of some new 1,4-disubstituted sulfonyl-1,2,3-triazoles and their antibacterial activity studies. *Med. Chem. Res.* 26 2190–2195. 10.1007/s00044-017-1926-6

[B71] Trizna E. Latypova L. Kurbangalieva A. Bogachev M. Kayumov A. (2016). 2(5*H*)-Furanone derivatives as inhibitors of staphylococcal biofilms. *BioNanoScience* 6 423–426. 10.1007/s12668-016-0258-1

[B72] Trizna E. Y. Khakimullina E. N. Latypova L. Z. Kurbangalieva A. R. Sharafutdinov I. S. Evtyugin V. G. (2015). Thio derivatives of 2(5*H*)-furanone as inhibitors against *Bacillus subtilis* biofilms. *Acta Naturae* 7 102–107 PMC4463419 26085951

[B73] Yujie L. Geng X. Huang Y. C. Li Y. Yang K. Y. Ye L. H. (2013). The Effect of brominated furanones on the formation of *Staphylococcus aureus* biofilm on PVC. *Cell Biochem. Biophys.* 67 1501–1505. 10.1007/s12013-013-9652-2 23979982

