# Supplementary material for: Antimicrobial Effects of Sulfonyl Derivative of 2(5H)-Furanone against Planktonic and Biofilm Associated Methicillin-Resistant and -Susceptible Staphylococcus aureus
Source: Front Microbiol. 2017 Nov 20;8:2246. doi: 10.3389/fmicb.2017.02246 (PMC5701942; doi:10.3389/fmicb.2017.02246)
Supplement: Supplementary file 1 [file Data_Sheet_1.pdf]

Supplementary Material

Antimicrobial effects of sulfonyl derivative of 2(5*H*)-furanone against planktonic and biofilm associated methicillin-resistant and -susceptible *Staphylococcus aureus*

Irshad S Sharafutdinov, Elena Y Trizna, Diana R Baydamshina, Regina R Sibgatullina, Alsu M Khabibrakhmanova, Liliya Z Latypova, Almira R Kurbangalieva, Elvira V Rozhina, Mareike Klinger-Strobel, Rawil F Fakhrullin, Mathias W Pletz, Mikhail I Bogachev, Airat R Kayumov*, Oliwia Makarewicz

*** Correspondence:** Airat R Kayumov, kairatr@yandex.ru

1 Chemistry

3,4-Dichloro-5-hydroxyfuran-2(5*H*)-one (mucochloric acid, **1**) (Shostka Chemical Reagents Plant, Ukraine) was recrystallized from water, mp 127°C. (1*R*,2*S*,5*R*)-(–)-Menthol (Acros Organics), 4-methylthiophenol (Alfa Aesar) were used without further purification. All solvents were purified and distilled by standard procedures. IR spectra of compounds **2**–**4** were recorded on a Bruker Tensor-27 spectrometer in a solid state (in Nujol). NMR spectra were measured on Bruker Avance III 400 spectrometer at 400.17 MHz (^{1}H) and 100.62 MHz (^{13}C) at 25°C using residual protonated solvent signals as the internal standard. Multiplicities are indicated as: s (singlet), d (doublet), ddd (doublet of doublets of doublets), septd (septet of doublets), m (multiplet). Analytical thin layer chromatography (TLC) was carried out on Sorbfil PTLC-AF-A-UF plates using dichloromethane as the eluent and UV light as the visualizing agent. Silica gel 60 (Acros, 70–230 mesh, 0.063–0.200 mm) was used for open column chromatography. The melting points were measured on an OptiMelt Stanford Research Systems MPA100 automated melting point apparatus and were not corrected. Optical rotation was measured on a Perkin-Elmer model 341 polarimeter in chloroform (concentration *c* is given as g/100 mL).

1.1 Synthetic procedures

1.1.1 Synthesis of furanone 2a

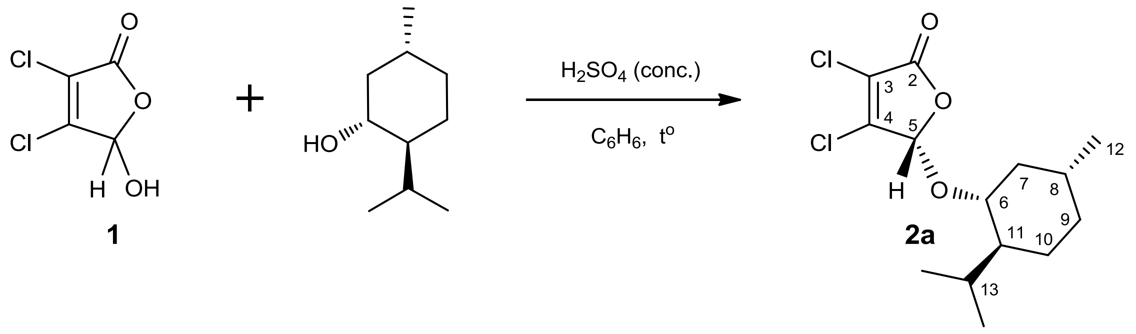

3,4-Dichloro-5(*S*)-[(1*R*,2*S*,5*R*)-2-isopropyl-5-methylcyclohexyloxy]-2(5*H*)-furanone (2a) was synthesized according to a slightly modified procedure generally following[1]. The solution of mucochloric acid (**1**) (4.00 g, 23.7 mmol), (1*R*,2*S*,5*R*)-menthol (3.70 g, 23.7 mmol), and concentrated sulfuric acid (0.13 mL) in benzene (70 mL) was heated at reflux for 13 h, with the water produced being removed by means of a Dean-Stark apparatus. After the completion of the reaction, the mixture was washed with 6% aqueous sodium bicarbonate (70 mL), the organic layer was evaporated to dryness under reduced pressure affording a solid colorless sample of **2** (6.33 g, 87%) as a 1:1 mixture of diastereomers **2a** and **2b** (measured by ^{1}H NMR spectroscopy).

Enantiomerically pure **2a** as white needles was obtained after two crystallizations from hexane. The combined mother liquors which were enriched with the **2b** isomer were next evaporated to dryness. The remaining solid was redissolved in benzene and heated at reflux for 11 hours in the presence of concentrated sulfuric acid (0.13 mL), and exhibited a mixture of the epimers, **2a**:**2b** = 1:1. The solution was washed with 6% aqueous sodium bicarbonate (70 mL), the organic layer was evaporated to dryness and the residue was crystallized twice from hexane to afford additional enantiomerically pure **2a** (combined yield 3.78 g, 52%), mp 113°C (mp 108–110°C ([1])). TLC R_f 0.70, $[\alpha]_D^{20}$ = +38.9 ($CHCl_3$, *c* = 1.0). IR, ν, cm^{-1}: 1770 (C=O), 1643 (C=$C_{lactone}$). ^{1}H NMR (acetone-*d*$_6$), δ, ppm: 0.86 (d, $^{3}J_{HH}$ = 7.0 Hz, 3H, CH_3 (*i*Pr)), 0.93 (d, $^{3}J_{HH}$ = 7.0 Hz, 3H, CH_3 (*i*Pr)), 0.94 (d, $^{3}J_{HH}$ = 6.5 Hz, 3H, CH_3, H^{12}), 0.82–1.14 (m, 3H, H^7, H^9, H^{10}), 1.31–1.40 (m, 1H, H^{11}), 1.41–1.56 (m, 1H, H^8), 1.64–1.73 (m, 2H, H^9, H^{10}), 2.19–2.27 (m, 1H, H^7), 2.32 (septd, $^{3}J_{HH}$ = 7.0 Hz, $^{3}J_{HH}$ = 2.7 Hz, 1H, H^{13}), 3.78 (ddd, $^{3}J_{HH}$ = 10.7 Hz, $^{3}J_{HH}$ = 4.4 Hz, 1H, H^6), 6.25 (s, 1H, H^5). ^{13}C{^{1}H} NMR (acetone-*d*$_6$), δ, ppm: 17.2, 22.1 (CH_3 (*i*Pr)), 23.4 (C^{12}), 24.6 (C^{10}), 27.1 (C^{13}), 33.1 (C^8), 35.7 (C^9), 44.1 (C^7), 49.9 (C^{11}), 85.4 (C^6), 104.2 (C^5), 125.0 (C^3), 150.0 (C^4), 164.8 (C^2). ^{1}H NMR ($CDCl_3$), δ, ppm: 0.81 (d, $^{3}J_{HH}$ = 7.0 Hz, 3H, CH_3 (*i*Pr)), 0.93 (d, $^{3}J_{HH}$ = 7.0 Hz, 3H, CH_3 (*i*Pr)), 0.94 (d, $^{3}J_{HH}$ = 6.5 Hz, 3H, CH_3, H^{12}), 0.83–1.16 (m, 3H, H^7, H^9, H^{10}), 1.32–1.48 (m, 2H, H^8, H^{11}), 1.63–1.73 (m, 2H, H^9, H^{10}), 2.19–2.31 (m, 2H, H^7, H^{13}), 3.58 (ddd, $^{3}J_{HH}$ = 10.7 Hz, $^{3}J_{HH}$ = 4.4 Hz, 1H, H^6), 5.81 (s, 1H, H^5). ^{13}C{^{1}H} NMR ($CDCl_3$), δ, ppm: 15.9, 20.8 (CH_3 (*i*Pr)), 22.1 (C^{12}), 23.0 (C^{10}), 25.3 (C^{13}), 31.6 (C^8), 33.9 (C^9), 42.1 (C^7), 48.0 (C^{11}), 84.5 (C^6), 102.2 (C^5), 124.0 (C^3), 147.6 (C^4), 163.5 (C^2).

1.1.2 Synthesis of thioether 3

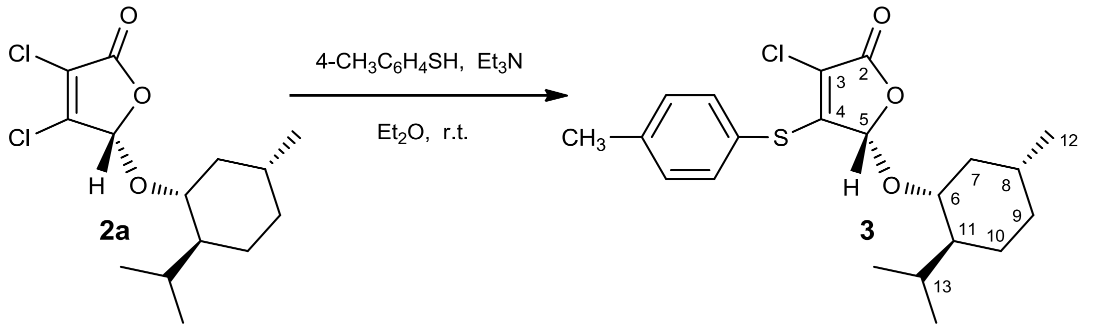

3-Chloro-5(*S*)-[(1*R*,2*S*,5*R*)-2-isopropyl-5-methylcyclohexyloxy]-4-[4-methylphenylsulfanyl]-2(5*H*)-furanone (3). To a solution of furanone **2a** (1.97 g, 6.4 mmol) in diethyl ether (20 mL) with intense stirring was added dropwise a solution of *p*-thiocresol (0.80 g, 6.4 mmol) in diethyl ether (5 mL), and a solution of triethylamine (0.90 ml, 6.4 mmol) in diethyl ether (5 mL). The reaction mixture was stirred at room temperature for 8 h, precipitated triethylamine hydrochloride was filtered out and washed with diethyl ether. The combined filtrates were evaporated to dryness and the

obtained solid residue was recrystallized from hexane forming colorless crystals yielding 2.15 g (85%), mp 116°C. TLC R_f 0.66, $[\alpha]_D^{20}$ = –122.7 ($CHCl_3$, c = 1.0). IR, ν, cm^{-1}: 1781 (C=O), 1643 ($C=C_{lactone}$), 1595, 1493 ($C=C_{arom}$). ^1H NMR ($CDCl_3$), δ, ppm: 0.65 (d, $^3J_{HH}$ = 7.0 Hz, 3H, CH_3 (*i*Pr)), 0.86 (d, $^3J_{HH}$ = 7.0 Hz, 3H, CH_3 (*i*Pr)), 0.90 (d, $^3J_{HH}$ = 6.6 Hz, 3H, CH_3, H^{12}), 0.75–1.10 (m, 3H, H^7, H^9, H^{10}), 1.22–1.32 (m, 1H, H^{11}), 1.30–1.42 (m, 1H, H^8), 1.58–1.67 (m, 2H, H^9, H^{10}), 1.95 (septd, $^3J_{HH}$ = 7.0 Hz, $^3J_{HH}$ = 2.5 Hz, 1H, H^{13}), 2.07–2.16 (m, 1H, H^7), 2.39 (s, 3H, CH_3 (*p*-Tol)), 3.45 (ddd, $^3J_{HH}$ = 10.7 Hz, $^3J_{HH}$ = 4.4 Hz, 1H, H^6), 5.82 (s, 1H, H^5), 7.20, 7.41 (AA'BB', $N = {}^3J_{AB} + {}^5J_{AB'}$ = 8.0 Hz, 4H, H_{arom}). $^{13}C\{^1H\}$ NMR ($CDCl_3$), δ, ppm: 15.8, 21.1 (CH_3 (*i*Pr)), 21.3 (CH_3 (*p*-Tol)), 22.1 (C^{12}), 22.8 (C^{10}), 25.0 (C^{13}), 31.7 (C^8), 33.9 (C^9), 42.2 (C^7), 48.0 (C^{11}), 82.8 (C^6), 101.7 (C^5), 120.1, 122.6, 130.2, 134.4, 140.4 (C^3, C_{arom}), 154.1 (C^4), 165.2 (C^2). $C_{21}H_{27}ClO_3S$ (394.96): calcd. C 63.86, H 6.89, Cl 8.98, S 8.12; found C 63.94, H 6.82, Cl 8.94, S 8.15.

1.1.3 Synthesis of sulfone 4

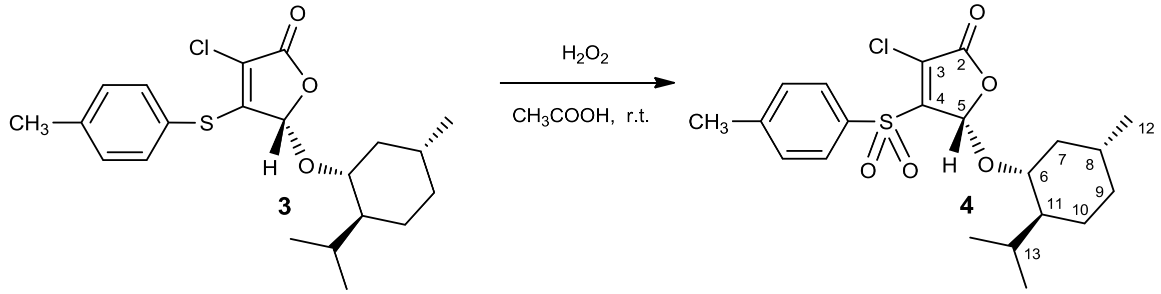

3-Chloro-5(*S*)-[(1*R*,2*S*,5*R*)-2-isopropyl-5-methylcyclohexyloxy]-4-[4-methylphenylsulfonyl]-2(5*H*)-furanone (4). Thioether **3** (0.71 g, 1.8 mmol) was dissolved in 15 mL of glacial acetic acid, 33% hydrogen peroxide (1.80 mL, 18.0 mmol) was added under stirring, and the mixture was stirred for 5 days at room temperature. When the reaction was complete, the mixture was evaporated to dryness, and the white solid residue was recrystallized from hexane forming colorless crystals yielding 0.53 g (70%), mp 105–106°C. TLC R_f 0.58, $[\alpha]_D^{20}$ = +132.9 ($CHCl_3$, c = 1.0). IR, ν, cm^{-1}: 1796 (C=O), 1619 ($C=C_{lactone}$), 1594 ($C=C_{arom}$), 1348 ($SO_{2\ asym}$), 1170 ($SO_{2\ sym}$). ^1H NMR ($CDCl_3$), δ, ppm: 0.85 (d, $^3J_{HH}$ = 6.9 Hz, 3H, CH_3 (*i*Pr)), 0.91 (d, $^3J_{HH}$ = 6.5 Hz, 3H, CH_3, H^{12}), 0.95 (d, $^3J_{HH}$ = 6.9 Hz, 3H, CH_3 (*i*Pr)), 0.74–1.10 (m, 3H, H^7, H^9, H^{10}), 1.23–1.33 (m, 1H, H^{11}), 1.34–1.48 (m, 1H, H^8), 1.62–1.73 (m, 2H, H^9, H^{10}), 2.12–2.20 (m, 1H, H^7), 2.41 (septd, $^3J_{HH}$ = 6.9 Hz, $^3J_{HH}$ = 1.9 Hz, 1H, H^{13}), 2.47 (s, 3H, CH_3 (*p*-Tol)), 3.71 (ddd, $^3J_{HH}$ = 10.7 Hz, $^3J_{HH}$ = 4.4 Hz, 1H, H^6), 6.28 (s, 1H, H^5), 7.39, 7.89 (AA'BB', $N = {}^3J_{AB} + {}^5J_{AB'}$ = 8.2 Hz, 4H, H_{arom}). $^{13}C\{^1H\}$ NMR ($CDCl_3$), δ, ppm: 15.7, 22.07 (CH_3 (*i*Pr)), 21.1 (C^{12}), 21.8 (CH_3 (*p*-Tol)), 22.6 (C^{10}), 24.6 (C^{13}), 31.6 (C^8), 33.8 (C^9), 42.1 (C^7), 48.2 (C^{11}), 83.6 (C^6), 101.6 (C^5), 128.7, 130.0, 132.6, 135.4, 146.7 (C^3, C_{arom}), 151.3 (C^4), 163.1 (C^2). ^1H NMR (DMSO-d_6), δ, ppm: 0.77 (d, $^3J_{HH}$ = 7.0 Hz, 3H, CH_3 (*i*Pr)), 0.84 (d, $^3J_{HH}$ = 7.0 Hz, 3H, CH_3 (*i*Pr)), 0.88 (d, $^3J_{HH}$ = 6.5 Hz, 3H, CH_3, H^{12}), 0.71–1.05 (m, 3H, H^7, H^9, H^{10}), 1.12–1.23 (m, 1H, H^{11}), 1.33–1.49 (m, 1H, H^8), 1.54–1.65 (m, 2H, H^9, H^{10}), 2.06–2.15 (m, 1H, H^7), 2.24 (septd, $^3J_{HH}$ = 7.0 Hz, $^3J_{HH}$ = 2.1 Hz, 1H, H^{13}), 2.43 (s, 3H, CH_3 (*p*-Tol)), 3.74 (ddd, $^3J_{HH}$ = 10.5 Hz, $^3J_{HH}$ = 4.4 Hz, 1H, H^6), 6.57 (s, 1H, H^5), 7.54, 7.87 (AA'BB', $N = {}^3J_{AB} + {}^5J_{AB'}$ = 8.3 Hz, 4H, H_{arom}). $^{13}C\{^1H\}$ NMR (DMSO-d_6), δ, ppm: 15.7, 21.0 (CH_3 (*i*Pr)), 21.3 (CH_3 (*p*-Tol)), 22.1 (C^{12}), 22.2 (C^{10}), 24.1

(C^{13}), 30.9 (C^{8}), 33.4 (C^{9}), 41.8 (C^{7}), 47.5 (C^{11}), 82.3 (C^{6}), 101.8 (C^{5}), 128.0, 130.3, 132.3, 135.3, 146.6 (C^{3}, $C_{arom.}$), 150.3 (C^{4}), 162.9 (C^{2}). $C_{21}H_{27}ClO_5S$ (426.95): calcd. C 59.08, H 6.37, Cl 8.30, S 7.51; found C 59.21, H 6.28, Cl 8.34, S 7.50.

1.2 ^{1}H and $^{13}C\{^{1}H\}$ NMR Spectra

a)

b)

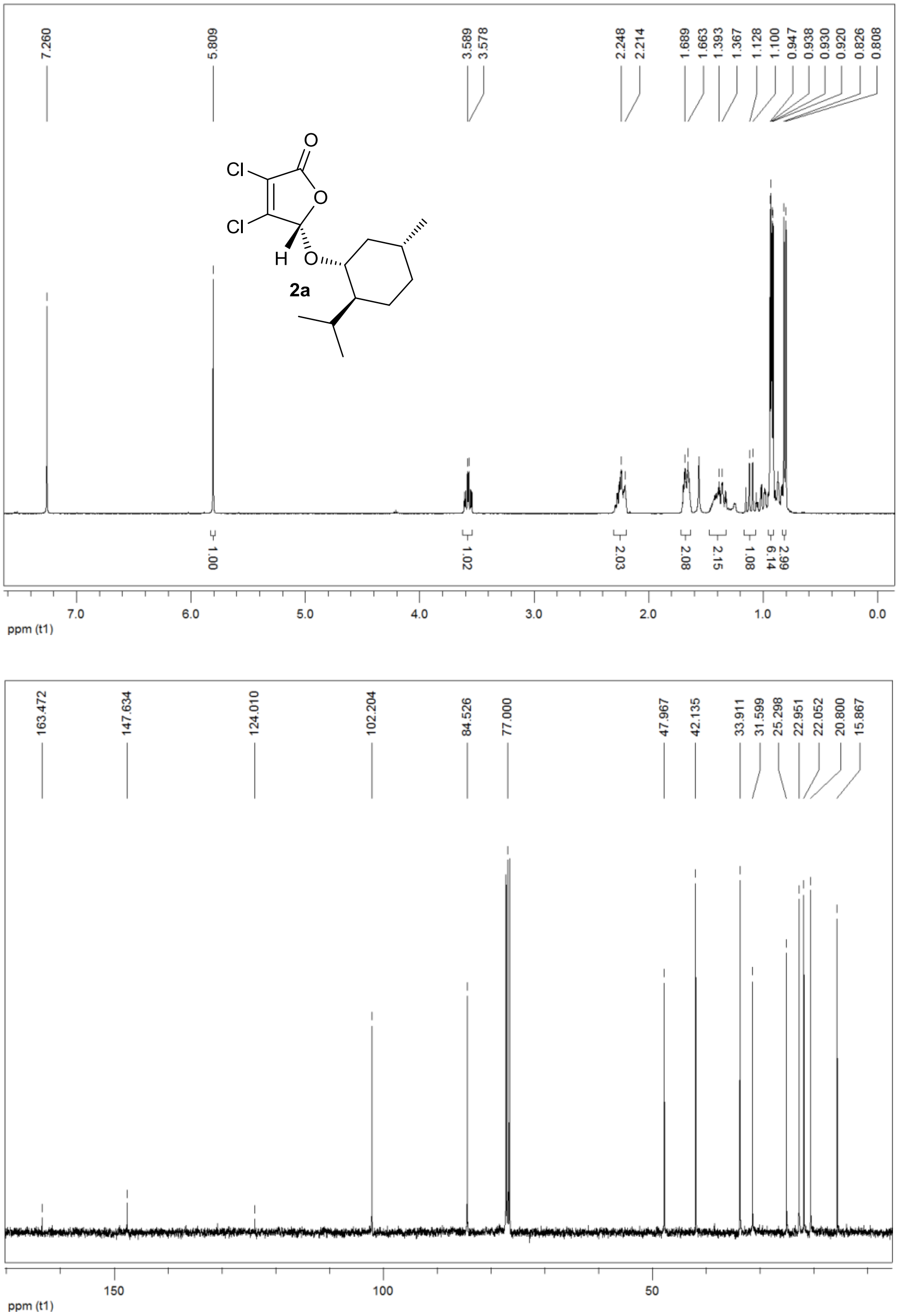

Figure S1. ^{1}H (a) and $^{13}C\{^{1}H\}$ (b) NMR spectra of compound **2a** ($CDCl_3$).

a)

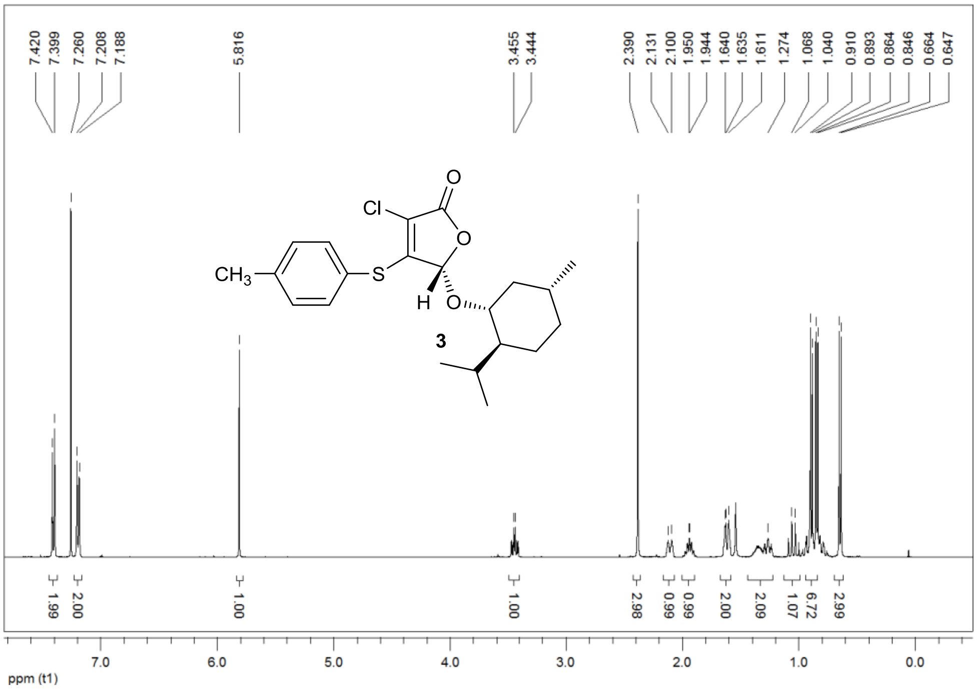

b)

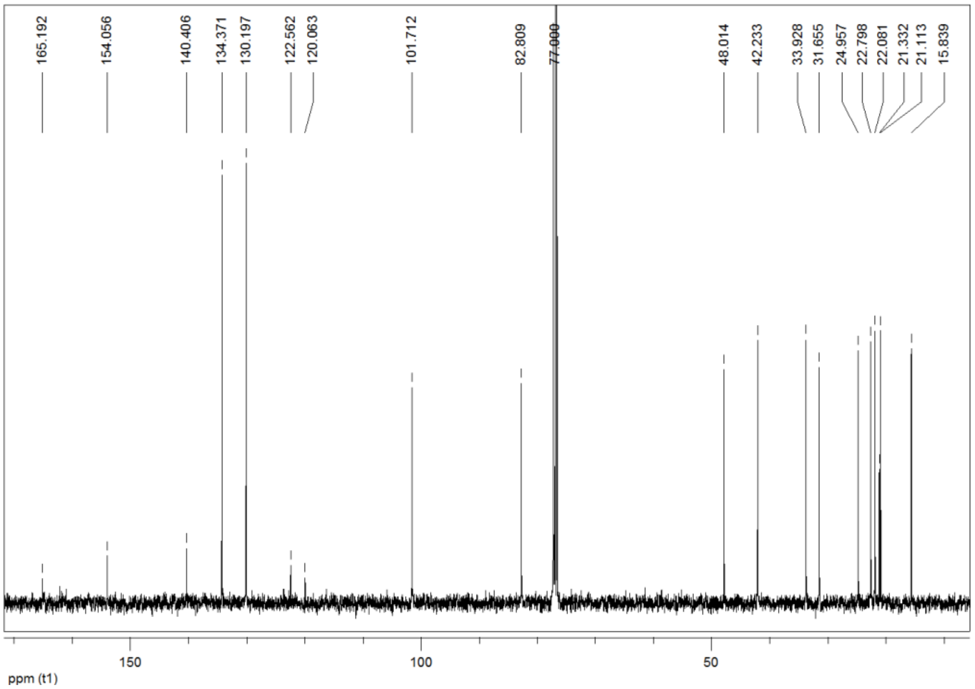

Figure S2. ^{1}H (a) and ^{13}C{^{1}H} (b) NMR spectra of compound **3** ($CDCl_3$).

a)

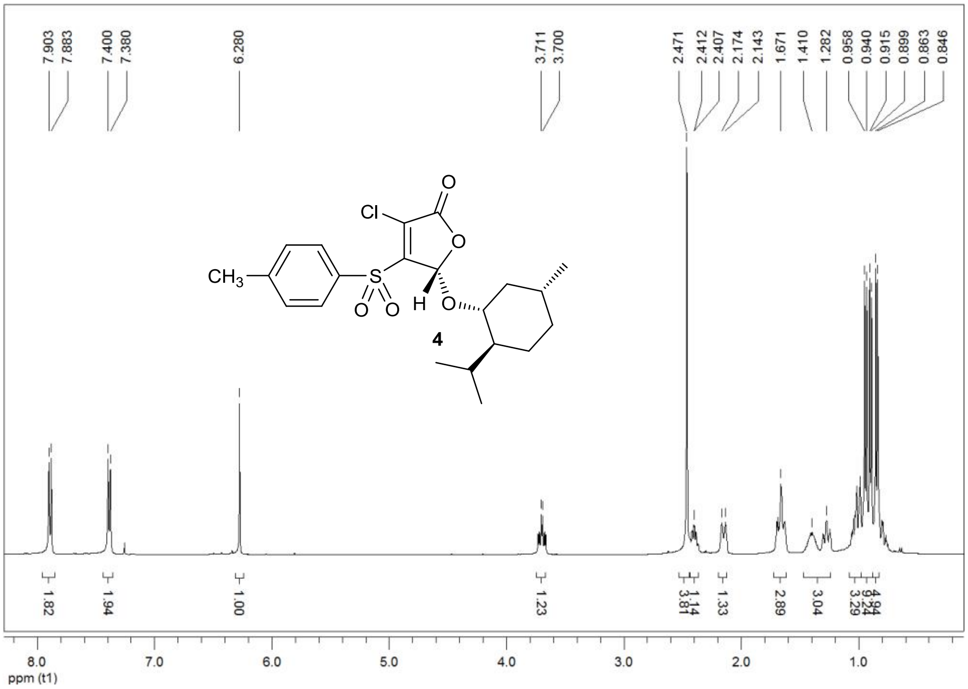

b)

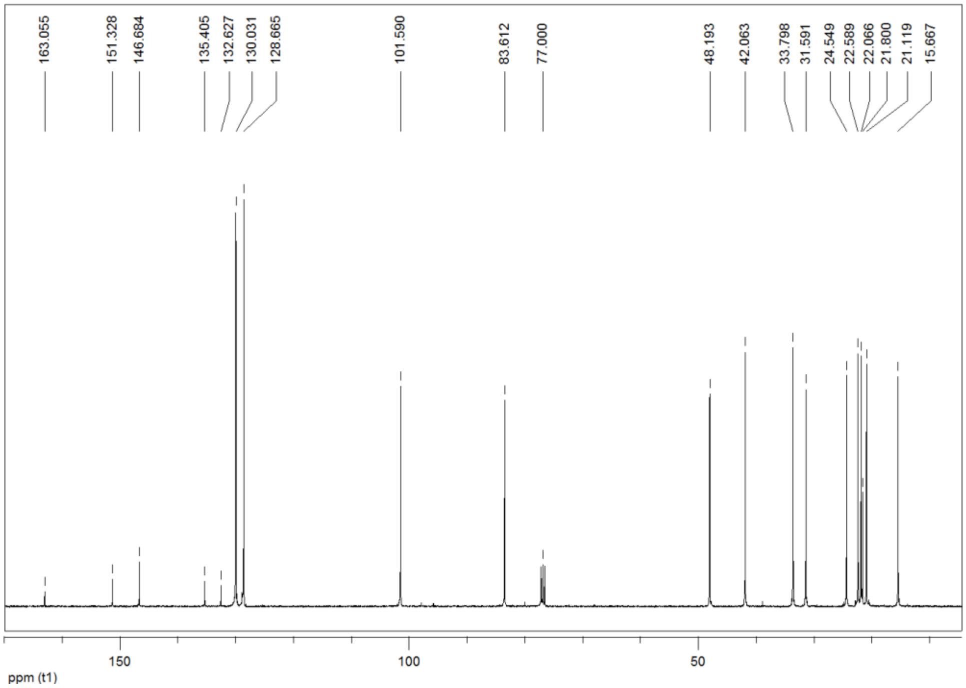

Figure S3. ^{1}H (a) and $^{13}C\{^{1}H\}$ (b) NMR spectra of compound **4** ($CDCl_3$).

1.3 IR Spectra

a)

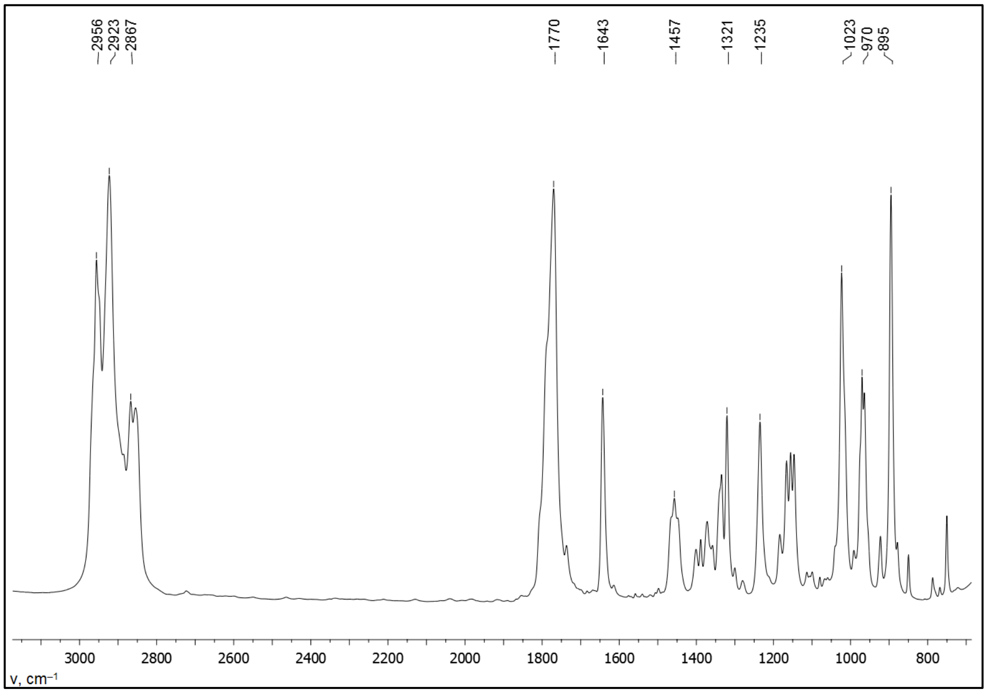

b)

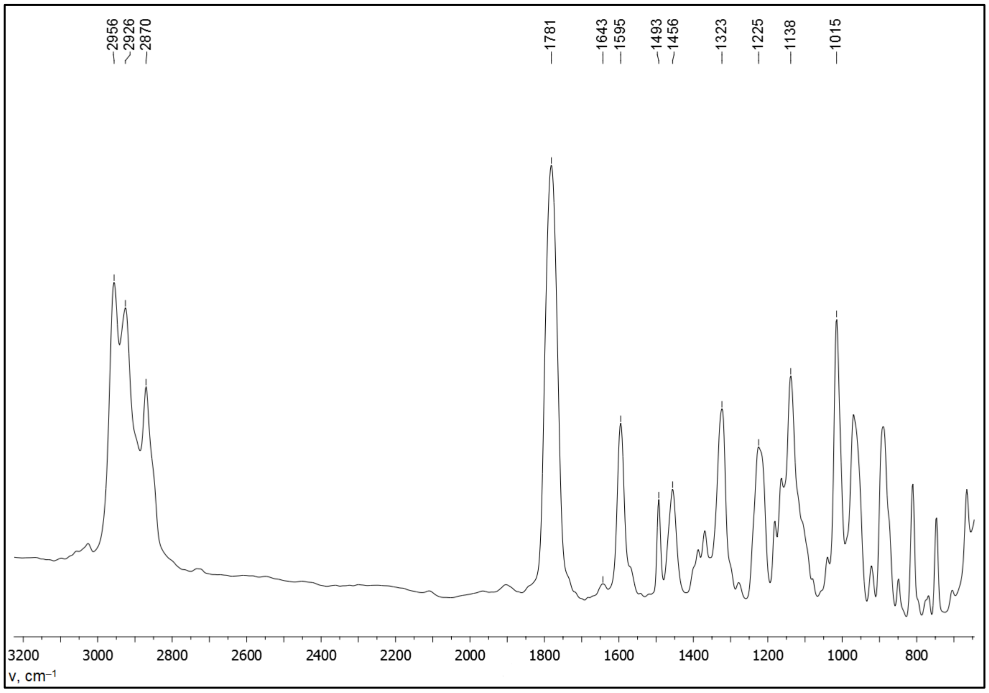

Figure S4. IR spectra of compounds **2a** (a) and **3** (b).

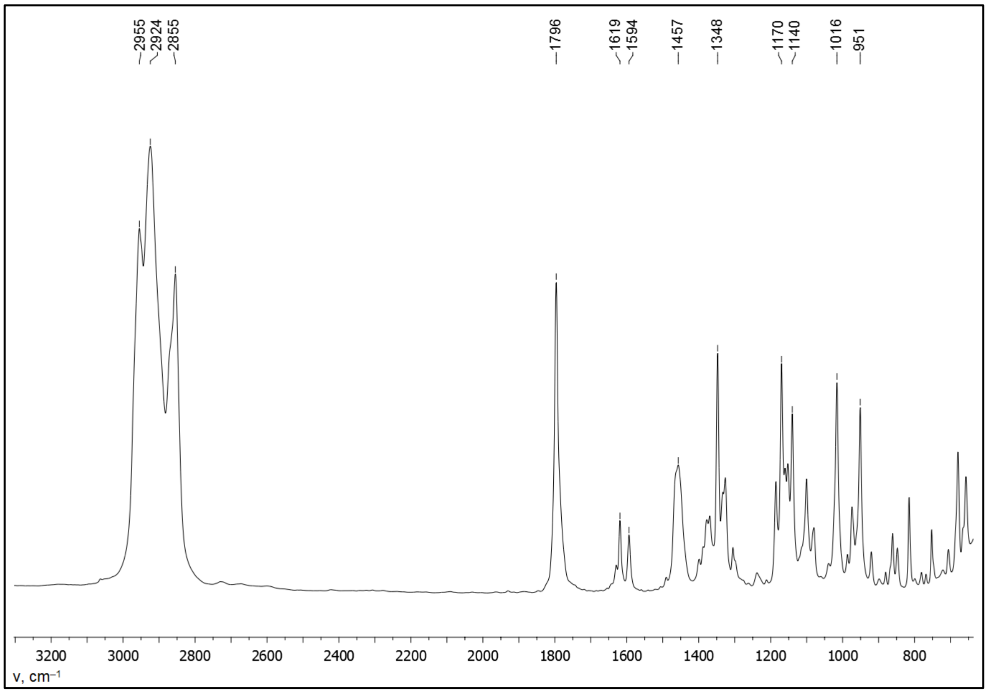

Figure S5. IR spectrum of compound **4**.

2 Biological data

Table S1. MIC, FICI and EC_{50} for **F105** and antibiotics on MRSA cells

	MIC, mg/L	ECOFF, mg/L	$FICI_{min}$	EC_{50}, mg/L
Gentamicin	0.13	2.00	0.31	0.61
Daptomycin	2.00	1.00	0.51	4.19
Rifampicin	< 0.03	0.03	0.75	3.01
Linezoild	2.00	4.00	0.50	1.27

Table S2. MBC of different antimicrobials on MSSA cells

	MBC, mg/L
Ampicillin	8
Gentamycin	4
Benzalkonium	2
**F105**	40

Table S3. Mutagenicity of **F105** evaluated by the Ames test without metabolic activation and expressed as excess of the revertant colonies number over the negative control. The compound was considered as mutagenic when the revertants number exceeded the negative control more than twofold.

Compound, mg/L	*S. typhimurium* strain		
	TA100	TA98	TA102
**F105**, 5	1.5±0.47	1.4±1.07	0.8±0.19
**F105**, 10	1.5±0.13	1.1±0.98	0.9±0.37
**F105**, 20	1.3±0.27	1.7±1.26	1.3±0.61
**F105**, 40	0.8±0.11	1.6±1.12	0.8±0.48
**F105**, 80	0.8±0.11	1.3±0.83	1.1±0.54
Negative control (DMSO)	1.00	1.00	1.00
NaN_3, 10	17.8±2.17	-	2.1±0.18
4-Nitro-o-phenylenediamine, 10	-	44.4±2.65	-

Table S4. Mutagenicity of **F105** evaluated by the Ames test with metabolic activation by S9 fraction and expressed as excess of the revertant colonies number over the negative control, fold. The compound was considered as mutagenic when the revertants number exceeded the negative control more than twofold.

Compound, mg/L	*S. typhimurium* strain		
	TA100	TA98	TA102
**F105**, 80	1.1±0.75	1.5±0.98	0.8±0.22
Negative control (DMSO)	1.0	1.0	1.0
2-aminoanthracene, 0.6	-	23.9±1.65	-
2-aminoanthracene, 8	3.0±0.65	-	-
Benzo(a)pyrene, 1	-	-	3.3±0.95

Table S5. Intracellular proteins with decreased amount after growth in the presence of **F105** (LC-MS data)

Protein	Score*		emPAI$		MW
	control	**F105**	control	**F105**	
DNA-directed RNA polymerase subunit beta~	2367	163	0,54	0,08	135
Methionine--tRNA ligase	150	137	0,34	0,16	74
Molecular chaperone GroEL	1206	74	1,12	0,13	57
Inosine-5-monophosphate dehydrogenase	742	825	0,41	0,23	52
Alkaline phosphatase	1000	100	1,47	0,32	51
Enolase	2727	1716	2,4	0,58	47
Branched-chain alpha-keto acid dehydrogenase subunit E2	3089	102	1,35	0,47	46
Thioredoxine reductase	1814	2206	1,44	0,49	36
Glycerophosphodiester phosphodiesterase	497	40	0,84	0,22	35
Fructose-1,6-bisphosphate aldolase	5923	149	0,92	0,24	33
30S ribosomal protein S3	2627	171	2,24	0,55	24
Fibrinogen-binding protein	90	27	1,27	0,32	12
Thioredoxin	1800	300	3,45	1,45	11

* The score for an MS/MS match is based on the calculated probability, *P*, that the observed match between the experimental data and the database sequence is a random event. The reported score is –10log(*P*)

$ The **E**xponentially **M**odified **P**rotein **A**bundance **I**ndex (emPAI) offers approximate, label-free, relative quantitation of the proteins in a mixture based on protein coverage by the peptide matches in a database search result (Ishihama, Y., et al., 2005)

Table S6. Intracellular proteins with increased amount after growth in the presence of **F105** (LC-MS data)

Protein	Score		emPAI		MW
	control	**F105**	control	**F105**	
3-hydroxyacyl-CoA dehydrogenase	67	421	0,14	0,67	84
1-pyrroline-5-carboxylate dehydrogenase	40	213	0,14	0,56	56
Phosphoribosylamine--glycine ligase	82	663	0,17	0,87	45
Glutamate dehydrogenase	171	90	0,17	0,6	45
Ornithine--oxo-acid aminotransferase	26	509	0,18	0,78	43
Heme peroxidase	92	2072	0,27	0,62	29
Transaldolase	26	506	0,15	0,74	25

* The score for an MS/MS match is based on the calculated probability, *P*, that the observed match between the experimental data and the database sequence is a random event. The reported score is –10log(*P*).

[$] The **E**xponentially **M**odified **P**rotein **A**bundance **I**ndex (emPAI) offers approximate, label-free, relative quantitation of the proteins in a mixture based on protein coverage by the peptide matches in a database search result (Ishihama, Y., et al., 2005)

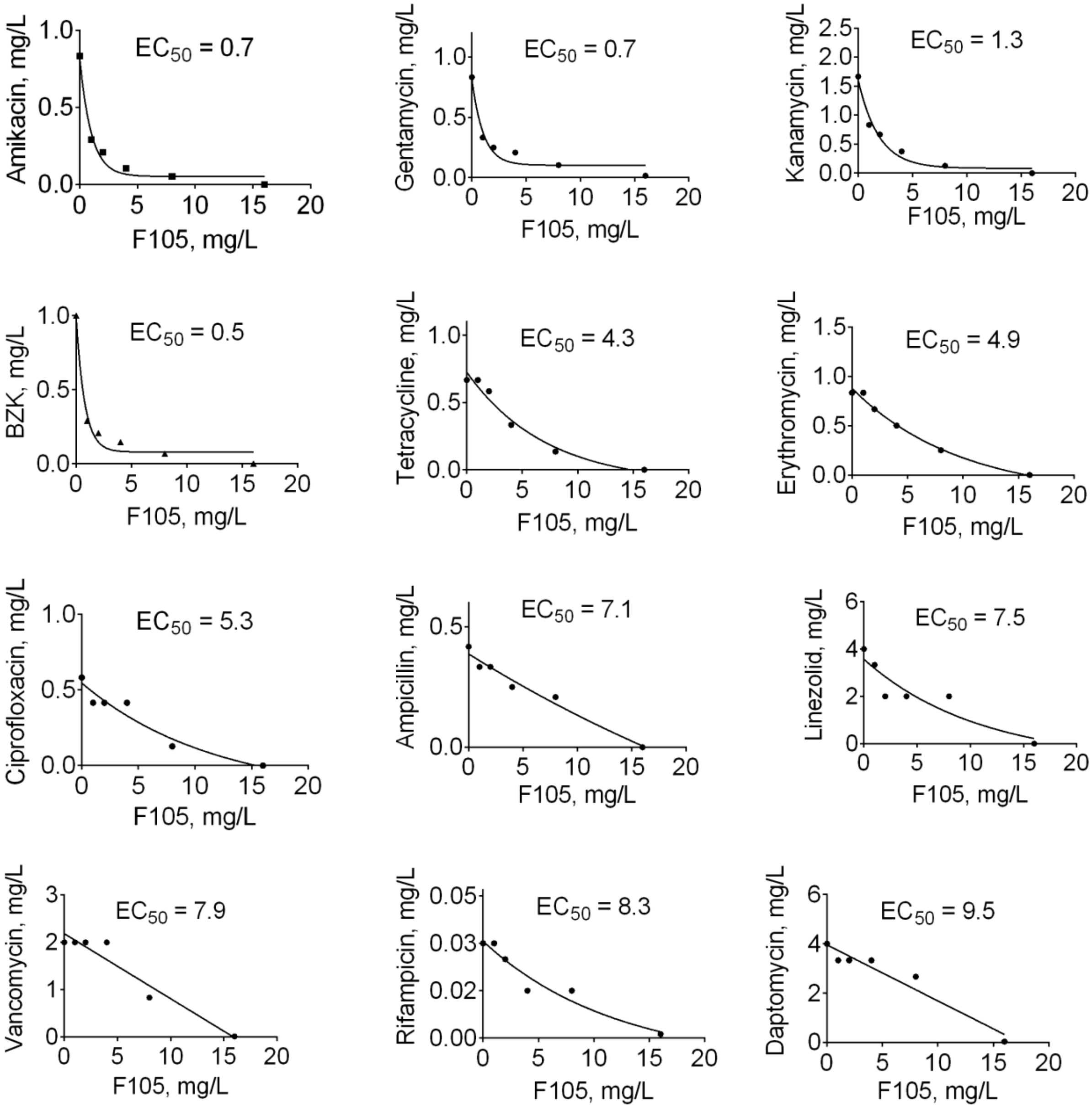

Figure S6. The synergy of **F105** with antibiotics from various classes, performed on MSSA. The effective concentration of **F105** (EC_{50}) is calculated as a **F105** concentration leading to double reduce of antibiotic's MIC.

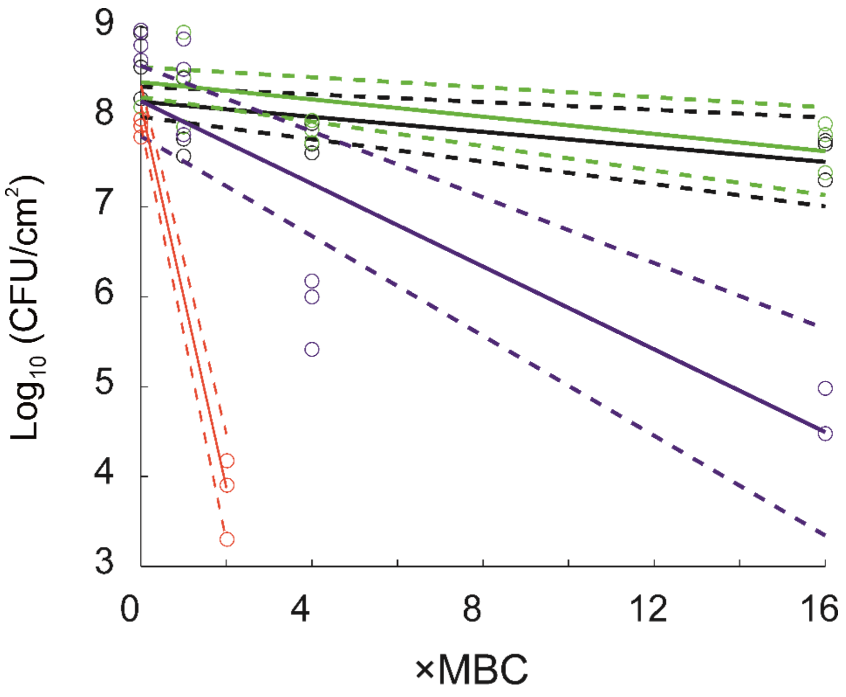

Figure S7. Dose-response curves for biofilm-embedded MSSA treated with different antimicrobials: Ampicillin (up to 16×MBC, black), Gentamicin (up to 16×MBC, green), Benzalkonium chloride (up to 16×MBC, blue) and **F105** (up to 4×MBC, red). Full lines denote regression lines, while dashed lines denote corresponding 95% confidence intervals.

3 Literature references

[1] Fenske, D., and Merzweiler, K. (1989). Synthesis of a new chiral phosphine ligand. *Zeitschrift Fur Naturforschung Section B-a Journal of Chemical Sciences* 44, 879-883.
